# Development of an open-source solution to facilitate the use of one-button wearables in experience sampling designs

**DOI:** 10.3758/s13428-023-02322-y

**Published:** 2024-01-17

**Authors:** Selina Volsa, David Lewetz, Vinka Mlakic, Chiara Bertagnoli, Samantha Hochstöger, Martina Rechl, Hannah Sertic, Bernad Batinic, Stefan Stieger

**Affiliations:** 1 Department of Psychology and Psychodynamics, Karl Landsteiner University of Health Sciences, Dr.-Karl-Dorrek-Straße 30, 3500 Krems an der Donau, Austria; 2 Department of Work, Organizational and Media Psychology, Johannes Kepler University Linz, Altenbergstraße 69, 4040 Linz, Austria

**Keywords:** Experience sampling method, Ecological momentary assessment, Wearables, Ambulatory assessment, Physical analogue scale

## Abstract

The experience sampling method (ESM) allows for a high degree of ecological validity compared to laboratory research, at the cost of greater effort for participants. It would therefore benefit from implementations that reduce participant effort. In the present paper, we introduce a screenless wrist-worn one-button wearable as an unobtrusive measurement method that can be employed in ESM designs. We developed an open-source Android application to make this commercially available wearable easily configurable and usable. Over the course of six pilot studies, we explored the technical viability (e.g., battery life, reliability of inputs) of this wearable. We compared data quality between wearables and smartphones in a within-subjects design, exploring both the input options of using the number of button presses as a Likert scale, as well as using the angle of the device as a Physical Analogue Scale. Assessments of Extraversion made with either of these methods were highly correlated to comparable assessments made with comparable methods on a smartphone (i.e., Likert scale or a Visual Analogue Scale, respectively). Furthermore, in a preregistered ESM field experiment (*N* = 134, 4 weeks), we compared compliance to real-life event triggers between wearable devices and smartphones. We found higher number**s** of logged events in the wearable group, indicating better adherence to the event-contingent scheduling. Overall, despite the device’s minimal capabilities and resulting limitations, one-button wearables can be beneficial for use in ESM designs.

**Supplementary Information:**

The online version contains supplementary material available at 10.3758/s13428-023-02322-y.

High ecological validity is a hallmark of field research (Mehl et al., 2014). This necessitates methods and instruments that facilitate optimal performance in these types of studies. Wrist-worn one-button wearables are a tool that has received little attention for use with in situ self-report methods (see van Berkel et al., 2017 for a review; Larsen et al., 2017; Stieger et al., 2020, 2022). These devices are versatile, allowing inputs on a Likert scale via a button, and analog inputs via a Physical Analogue Scale (PAS) (Stieger et al., 2020) using an accelerometer. They are especially convenient for active in situ self-tracking of frequent events. They are low effort and designed to reduce participant burden and thereby increase data quality. However, due to their lack of adoption, little is known about their reliability and usefulness. In this paper, we aim to validate one-button wearables in self-report research.

## The experience sampling method

As a longitudinal in situ self-report method, the experience sampling method (ESM) allows the generation of naturalistic data of high external validity (Larson & Csikszentmihalyi, 1983). This method is also commonly referred to as ecological momentary assessment (EMA) or ambulatory assessment (AA). In ESM designs, participants are required to fill out questionnaires that are either scheduled for certain times (Mehl et al., 2014) as indicated by the device, either at pseudo-random time points (signal contingent) or regularly at specific times (interval contingent), or as a result of an event occurring in the participants’ everyday life (event contingent). Scheduling occurs through notifications, also referred to as ‘bings’.

ESM designs place a higher burden on participants than laboratory experiments or surveys because ESM requires participants to regularly interrupt their daily routines for data entry, over an extended period. Managing this burden is thus essential, as ESM designs with high burden can lead to reduced data quality, more missing data, and more frequent and earlier dropout (e.g., Eisele et al., 2022; Fuller-Tyszkiewicz et al., 2013; Klasnja et al., 2008). Due to the high rates of smartphone use in the general public (https://www.statista.com/statistics/330695/number-of-smartphone-users-worldwide/), smartphone apps on the participant's personal devices, instead of carrying an additional device, are often used to reduce participants burden in an ESM study (van Berkel et al., 2017). However, several other systems of various levels of specialization have been proposed to alleviate participant burden. These include devices physically situated in a participant’s environment and operated via touch or button inputs. For example, Vega et al. (2018) explored several prototypes of devices to allow patients to report symptoms of Parkinson’s disease, including physical buttons fixed to a sheet of paper, and found that paper diaries had the highest compliance. Heed, a device developed by Paruthi et al. (2018), was specifically designed as a situated self-reporting device. Heed devices can be placed in a space the user visits frequently, are associated with individual ESM items, and can prompt the participant for an interaction via a flashing LED. Response options are printed just inside the circular, touch-sensitive rim of the Heed device, a few centimeters in diameter, which participants use to interact with the Heed device.

Another general approach to minimizing the burden is to minimize the interaction itself by using microinteractions (Ashbrook, 2010), which are interactions that take 4 s or less to complete. This short time includes both access time (i.e., the time required to retrieve and activate the device), and the usage time (i.e., the time required for the intended interaction with the device). Microinteractions mean that usually only a single item can be answered on a single measurement occasion; however, the number of occasions can be increased without increasing the overall burden. This use of microinteractions has shown to be beneficial for compliance rates when used on smartwatches (Intille et al., 2016; Ponnada et al., 2017).

However, there is mixed evidence regarding the use of microinteractions on smartphones. Chan et al. (2018) found good compliance and low burden when an item was displayed on a smartphone’s lock screen, which could be answered with the same gesture to unlock the device (e.g., swipe). This reduces perceived access time, as participants would already have to retrieve their phone and swipe to unlock it. The drawback of this design is that the sampling time is not set by a pseudo-randomly timed bing, but by the participants themselves. On the other hand, using bings for microinteraction-based ESM is also problematic, because the access time for smartphones takes up a considerable portion of the interaction. This was indicated by Ponnada et al. (2017), where most participants using microinteraction-based ESM on smartphones dropped out shortly after the study began, reporting increased burden as a reason for drop-out. Considering this, wrist-worn wearables, which offer minimal access time, may be a good alternative for ESM.

## Wearables

We define a wearable in this context as an electronic device that can be worn on the body. While there are many different methods, a common form factor is a wrist-worn device, similar to a watch. Smartwatches have already been used in ESM research (e.g., Hernandez et al., 2016; Intille et al., 2016; Laborde et al., 2021; Ponnada et al., 2017, 2022). Software solutions for implementing ESM on smartwatches are also available (Khanshan et al., 2021; Volsa et al., 2022), yet rare. Past research indicates that while microinteraction-based ESM designs on smartwatches might produce higher perceived burden in terms of feelings of interruption than traditional ESM on smartphones, this approach also results in higher compliance (Intille et al., 2016). Furthermore, as mentioned above, smartwatches typically produce acceptable burden in the context of microinteractions, while use of smartphones has sometimes failed due to participants’ perception of excessive burden (Ponnada et al., 2017). This suggests that, at least in the context of microinteractions, the convenience of a wrist-worn wearable might alleviate participant burden.

One-button wearables are similar to smartwatches but have received little attention in the ESM field so far (Larsen et al., 2017; Stieger et al., 2020, 2022). While their interfaces are limited, one-button wearables lend themselves well to microinteractions since they involve short and simple interactions, which are likely to minimize burden. One-button wearables also benefit from long battery life, which minimizes the need for maintenance. These features make one-button wearables a solid candidate for usage in an ESM study design.

**Fig. 1:**
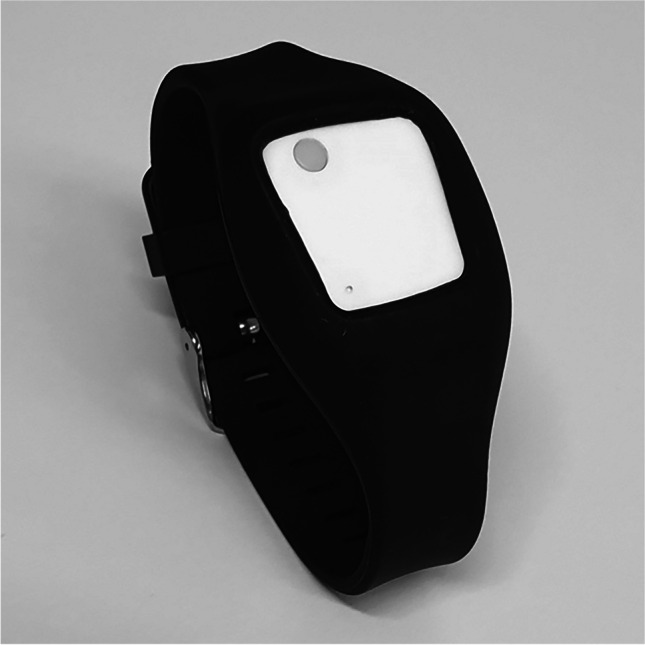
The MetaMotionR wearable in its wrist band

For the following studies, we used MetaMotionR devices by Mbientlab (shown in Fig. 1, see https://mbientlab.com/documentation/ for device specifications). These boards (built around an ARM 32-bit processor) come with fitting cases and rubber wrist bands and are commercially available through retail. Their features include an acceleration sensor, a tactile button, a coin vibration motor, and a red, blue, and green light-emitting diode (RBG-LED). One-button wearables can provide options for user input and feedback. Furthermore, they have an in-built program memory, 64 kB RAM, a timer (for exact time measurements), and a 60-mAh lithium polymer battery which can be charged with a micro-USB cable.

To make these devices easily usable for ESM research, we created an open-source application for Android smartphones. All communication between the smartphones and wearables is via Bluetooth. It is important to note that the wearable generally operates without the need for a connection to a smartphone; i.e., the smartphone is only necessary to program the wearable and to download the data from the wearable after data collection is over. The application can be downloaded from the Google Play Store (https://play.google.com/store/apps/details?id=at.jodlidev.metawear.study), and the source code is available on GitHub (https://github.com/KL-Psychological-Methodology/ESM-Board-Admin).

**Fig. 2:**
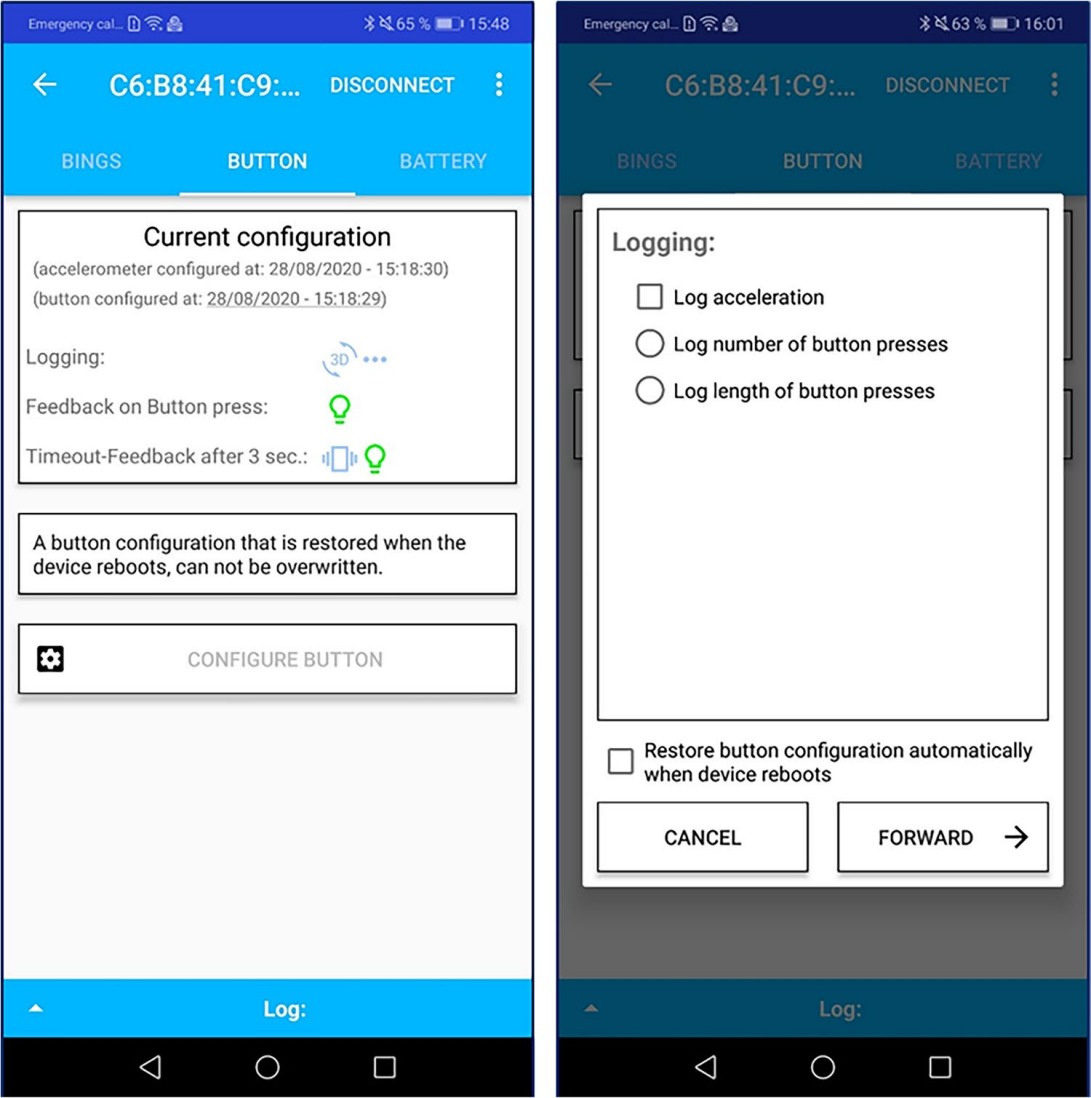
Screenshots of button configuration pane (*left*) and button logging configuration screen (*right*)

The input behavior of the wearable can also be configured via the smartphone application; i.e., how it behaves, and what is logged when the button is pressed. The device can either log the duration of presses or the number of consecutive presses. The latter counts the number of button presses that occur no more than 3 s apart from each other (i.e., if a press occurs less than 3 s after the last press, the counter is incremented, and the 3-s timer is reset; otherwise, the current count is logged). Counting of consecutive presses can be recorded using a sequential Likert scale. In addition to a mandatory button press parameter, the device can also optionally log acceleration. Acceleration data can be used to calculate the device’s orientation in 3D space, as explained in more detail below in the section regarding the PAS. Figure 2 shows screenshots of the button behavior overview as well as the logging options.

**Fig. 3:**
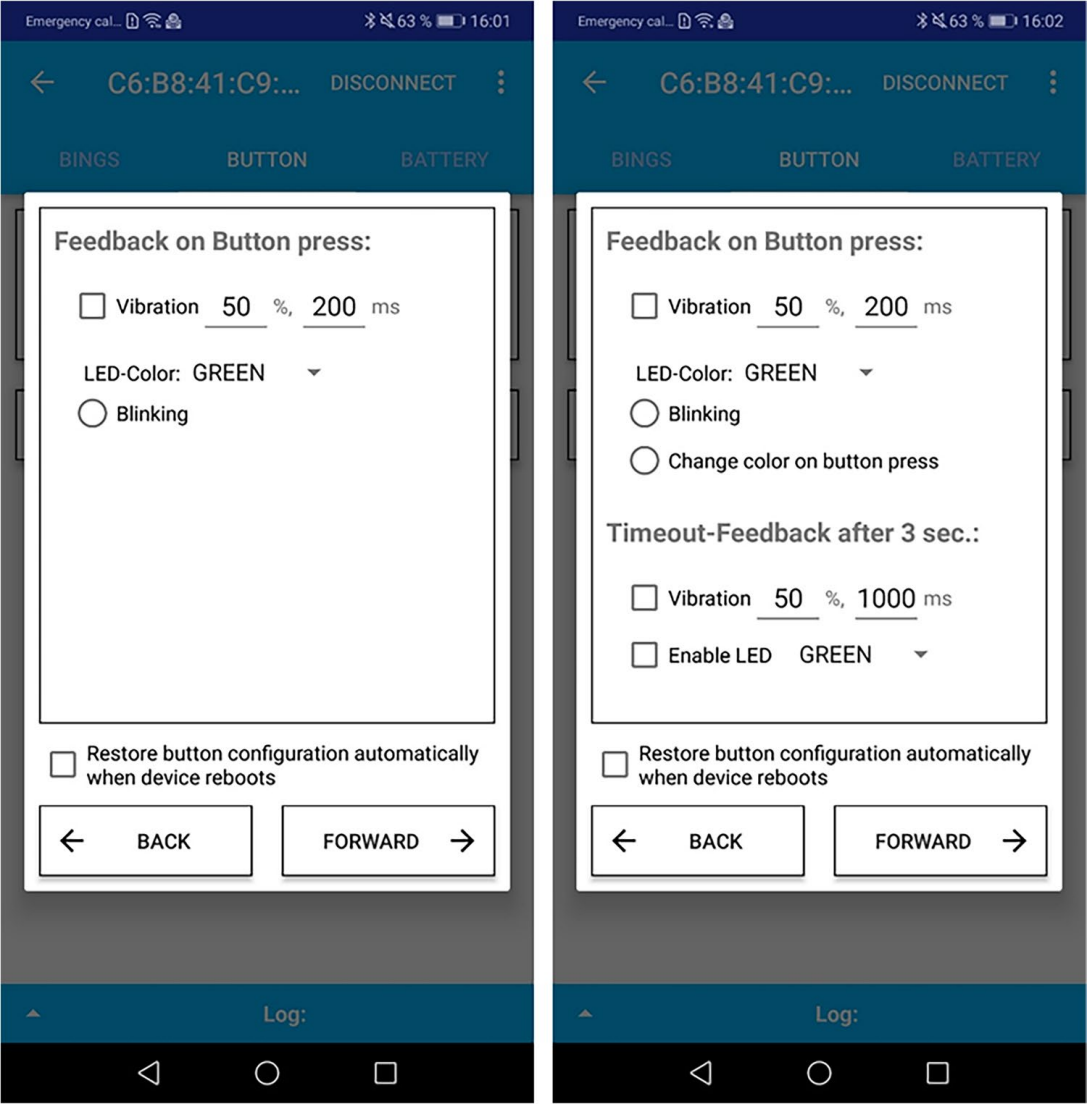
Screenshots of different button feedback options: for duration logging (*left*) and for count logging (*right*)

The application can also configure the feedback behavior for each button press. Each device reaction (i.e., user feedback to a button press) activates the device’s LED (in one of the colors red, green, or blue) and optionally activates the vibration motor. For the latter, the user can configure the duration and intensity of the vibration (Fig. 3, left). If the device is configured to log the button press count, then there are two additional configurable options (Fig. 3, right). One is for the device to cycle the LED color in a predictable pattern with each button press (i.e., green, blue, red, green) instead of one single color. The other option is to configure timeout feedback (with similar options to the button feedback), which occurs at the close of the 3-s window for consecutive button presses.

**Fig. 4:**
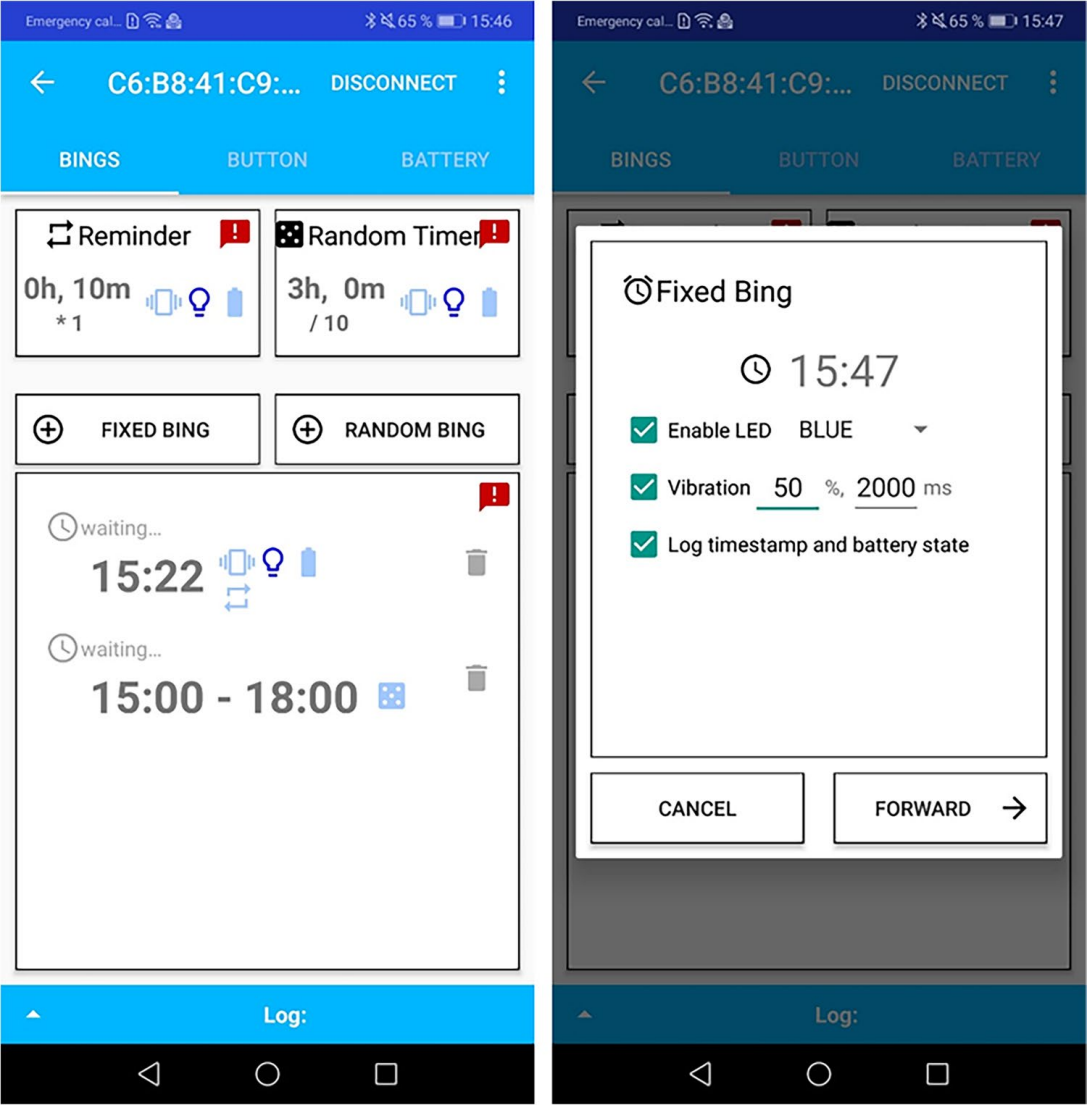
Screenshots of the bing configuration overview pane (*left*) and configuration options for a fixed bing (*right*)

Beyond the button, the application also has several options to configure bings. With one-button wearables, bings have the same feedback options as button presses (i.e., flashing the LED, activating the vibration motor). Furthermore, a bing can be set to log the timestamp of occurrence alongside the current battery percentage. Fixed bings are configured to occur daily at a specific time. Figure 4 shows screenshots of the bing overview and configuration options of a fixed bing. In addition, the application also has options to create pseudo-random bings. These are configured to occur in a set time frame. Due to device limitations, only one random time frame can be set, which can, however, be used for multiple bings. For example, it is possible to have one bing occur between 1:00 p.m. and 2:00 p.m. and another between 3:00 p.m. and 4:00 p.m., as these both have a 1-h time window. Overall, the device can store around five timers. This maximum is dependent on the specific type of timers used, as both pseudo-random bings and reminders (see below) internally require an additional timer. The mentioned maximum of five timers is the case for pseudo-random bings including reminders. The application also has options for reminders (i.e., if no reaction to the initial bing occurs), which can be configured to occur in set intervals and for a set number of times and are interrupted by button inputs. For example, a scheduled reminder will not occur if there is a button pressed beforehand. Due to technical limitations, there can only be one reminder configuration, but reminders can be reused across bings (i.e., all reminders share the same interval time and number of occurrences).

The aforementioned features are those which are likely most commonly used in ESM studies. However, the application has some additional features, such as the capability to restore parts of the configuration after a reboot. See the supplement for additional details and screenshots concerning the configuration application.

## Physical Analogue Scale (PAS)

With the limited input options of one-button wearables, it is important to make use of any information available to expand the device’s capabilities. One source of information is the accelerometer, which can be utilized to infer the device’s angle, and, thereby, the angle of the participant’s lower arm relative to the ground plane. This angle can be recorded using a PAS (Stieger et al., 2020, 2022). By assigning one scale-end to the horizontal position of the lower arm (0°) and the other to the upright vertical position (90°), participants can indicate a value in a gradual fashion, similar to a Visual Analogue Scale (VAS).

This angle calculation makes several assumptions. When resting in an (approximate) inertial system, the only acceleration it will measure is the earth’s gravitation. By analyzing the components of this vector, the angle between the wearable’s axes can be calculated. When the wearable is worn on the wrist, the *y*-axis will be parallel to the axis of the lower arm. This relation will not change, regardless of how the wearable is rotated around the wrist. This angle can be calculated using equation 1, where ${\uptheta }^{\prime}$ denotes the angle in degrees, and *x*, *y*, and *z* denote the components of the measured acceleration.

1 $${\theta }^{\mathrm{^{\prime}}}=atan\left(\frac{y}{\sqrt{{x}^{2}+{z}^{2}}}\right)\times \frac{180}{\pi }$$

This calculation is done automatically in the Android application mentioned above, meaning that the logfiles contain angles for all axes, and the resulting acceleration log data will directly contain the PAS values.

The maximum value of the scale can also be set in this equation. The factor in the numerator of the last term is twice the maximum value. In Eq. (1), this is set to 180 for angles, so the scale ranges from 0° (horizontal) to 90° (vertical). However, this value could also be set to 200 to create a scale from 0 to 100, comparable to some VAS implementations.

The sign of this angle is dependent on how the wearable is worn (i.e., just how a watch could also be worn with the clockface appearing upright or upside down), as well as which arm is used (i.e., left- or right-handed). However, the orientation around the wrist does not impact the calculation. Participants may wear the device on the back of their wrist, the inside, or any other position around the wrist. As long as each participant wears the device in a consistent location, the sign will be consistent.

One important note regarding this calculation is that it only gives the elevation angle utilizing a spherical coordinate system. Therefore, while PAS values below the lower scale end (i.e., below horizontal) can be distinguished from a value of the same angle above the lower end, values above the upper end (i.e., with the arm angled beyond vertical) will be mirrored back and are indistinguishable from values of the same angle below the upper end. Theoretically, it would be possible to infer the absolute orientation of the wearable; however, the radial orientation of the wearable on the wrist is uncertain, as this is dependent on where on the wrist the wearable is located (i.e., inside or outside of the wrist) and how the wrist is angled (e.g., angled in a way that improves button access). For this reason, it is important to record on which arm participants wore the wearable, so the affected data sets can be mirrored appropriately. Furthermore, participants must be instructed to keep both the arm and the orientation of the device on the wrist consistent throughout the duration of the study.

## Present studies

The aim of the present series of studies was to evaluate the application of one-button wearables within ESM studies. First, we conducted six pilot studies. Pilot studies 1 and 2 were concerned with the duration of battery life, with pilot study 1 assessing relatively new devices and pilot study 2 assessing devices of the same batch 2 years later, to account for aged batteries. Pilot study 3 assessed whether the device is prone to accidental button inputs. Pilot studies 4 and 5 assessed the variation in angle measurements that are necessary for the PAS. Pilot study 6 assessed the accuracy of user-estimated angle measurements. We further compared the same measurements performed using Likert scales on the wearable (i.e., the number of button presses represent the position on the Likert-type scale) and Likert scales used on a smartphone. Similarly, we compared PAS measurements done on the wearable to VAS measurements done on a smartphone within participants.

Finally, we performed a large study (*N* = 134; 28 days, *k* = 3045 data points) using mainly event-scheduled sampling to compare event-related compliance between the use of wearables and smartphones in a between-subjects experimental design. The study was designed to answer the following research question: are wearables beneficial for data quality (e.g., fewer missing data) compared to using a smartphone for data collection?

## Pilot study 1: Battery test 1

A key aspect of the use of one-button wearables for ESM is the reduction of participant burden. One aspect of burden is the requirement for maintenance. An example of this issue is demonstrated by Hernandez et al. (2016), whereby participants had more trouble keeping smartwatches charged than smartphones. While the device used in this study has a small battery (60 mAh), the very low power required to operate the wearable results in overall long battery life. In order to empirically analyze the device’s battery life, we performed a pilot study.

### Method

#### Materials

We tested the battery life of six randomly selected wearables (out of a pool of 170 wearables), with the goal of acquiring an estimate of a baseline battery run-time. These wearables were fully charged before the start of the test.

#### Wearable configuration

All wearables were configured to trigger a bing once a day using a pseudo-random timer, and once a day using a fixed timer. For each bing, the wearables would turn on their LED and vibrate. Reminders were not used. While this configuration is less complex than one that would be used in a typical ESM design, it is similar to the configuration used in the main study and can help to establish expectations for baseline battery life (as devices are always on, and functions like Bluetooth continuously consume power).

For three of the wearables (numbers 1, 3, and 6), the study conductor reacted to bings with a button press to create a log; however, this was only done when the study conductor was present, resulting in 140 reactions to 257 bings (54.4%). The study conductor did not react to bings on the other three wearables. This was done to assess whether or not active logging would influence the devices’ battery life.

One wearable (number 1) experienced a software error that caused the LED to stay turned on after a bing, only resetting with the next bing. This software error has since been fixed.

### Results

**Fig. 5:**
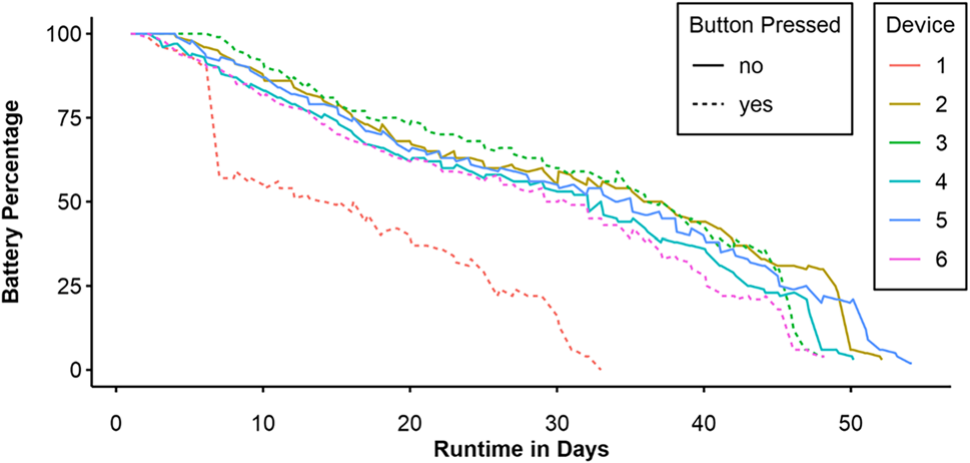
Battery charge (in percent) over time from pilot study 1

Runtime was measured as the time between the first and last recorded bing, with the first entry generated at the time of configuring the device. The overall mean runtime was *M* = 46.62 days (*SD* = 7.54). Table 1 shows the runtime of the individual wearables. However, as mentioned above, one wearable showed anomalous behavior during one bing. Figure 5 shows the discharging curve of the logged battery percentage over the wearables’ runtime. The mentioned anomaly is noticeable in Fig. 5 in the curve for wearable 1 toward the end of the first week. Excluding this device results in a mean runtime of *M* = 49.54 days (*SD* = 2.60).

**Table 1:** Battery runtime per wearable in days from pilot study 1

Wearable no.	1^ab^	2	3^a^	4	5	6^a^
Runtime	31.99	51.11	47.10	49.17	53.11	47.20

^a^ Devices for which the button was pressed. ^b^ Device in which the LED anomaly occurred once

### Discussion

While one device behaved anomalously due to a software bug, causing increased battery drain, the other devices experiencing more typical energy demand did not differ greatly in their runtime, with a standard deviation of less than 3 days. The two normally behaving devices that received button presses showed descriptively lower battery life than the other wearables. However, this difference is comparatively small, and all wearables 2–6 showed practically similar performance.

## Pilot study 2: Battery test 2

Lithium-ion batteries are used in the discussed wearable devices, yet they suffer from aging effects, reducing their capacity over time, dependent on use (Vetter et al., 2005). Due to this expected decline in capacity, we chose to repeat the battery test from pilot study 1 after approximately 2 years to gain a longer-term view of the batteries’ behavior.

### Method

The method was identical to that of pilot study 1, with the exception that no reaction presses to bings were performed. We used a separate batch of six randomly selected wearables from our pool of devices. All devices in that pool had been charged at regular intervals over a 2-year period to prevent the batteries from completely discharging. Due to the relatively long battery life observed in pilot study 1, devices that were actively used in other studies were not actively charged more often, resulting in the number of charge cycles across devices being comparable.

### Results

**Fig. 6:**
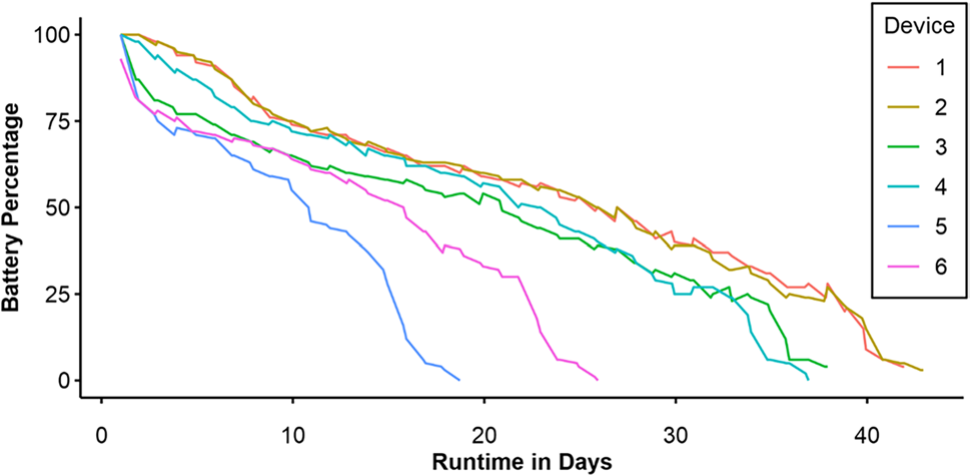
Battery charge (in percent) over time from pilot study 2

Figure 6 shows the trajectory of logged battery percentage over the wearables’ runtime. Despite our efforts to make sure all wearables were fully charged, the plot shows that the first data point of wearable 6 is slightly below 100%. Table 2 shows runtimes of this battery test in days. The average runtime was high, with a mean of 33.07 days, but with a higher variation in the overall runtime across wearables (*SD* = 9.65) compared to pilot study 1.

**Table 2:** Battery runtime per wearable in days from pilot study 2

Wearable no.	1	2	3	4	5	6
Runtime	40.94	41.94	36.93	35.93	17.72	24.93

### Discussion

The data show a decreased capacity of older batteries compared to the newer batteries in pilot study 1. Not only has the overall runtime decreased, but the discharge patterns are heterogenous, with variability between devices being larger than in the previous pilot study 1. This can most likely be attributed to some wearables having been used more than others, or some being stored at optimal charge for longer. Still, the batteries in all devices in this sample exceeded 2 weeks runtime, suggesting that devices with older batteries remain usable without the need to recharge regularly.

Both pilot studies 1 and 2 used 2 bings per day, which is sufficient for a range of ESM designs. However, some designs require a greater number of bings (e.g., compare observations per day in aan het Rot et al., 2012). A third battery test (available in the online supplement) suggests that a higher count of bings and reminders could diminish battery life, but remain in a usable range. Devices were tested using five bings per day, with five reminders per bing, consistently exceeded a runtime of 20 days.

## Pilot study 3: Assessment of accidental button presses

Because the wearable is always operational and does not have to be unlocked like a smartphone, the wearable’s button might occasionally be pressed unintentionally (e.g., while asleep). To explore the possibility of unintentional button presses, we conducted a 1-week ESM study by instructing participants not to press the button altogether.

### Method

Eight participants wore wearables for seven complete consecutive days, including at night. Participants were from a convenience sample and were balanced in gender (four female, four male). Their average age was 31.88 years (range, 26–45, *SD* = 6.49). Participants were predominantly right-handed (seven right-handed, one left-handed), and most wore the wearable on their left wrist (six left, two right). Wearables were only removed for showers or similar activities, as the devices are not waterproof. All wearables were configured to register and log button presses. The button was pressed on each device before and after the study duration to ensure that the devices were working and would indeed register button presses. Participants were instructed not to press the button during the study duration (i.e., 1-week field phase) and were further instructed to note the number of intentional (but unwanted) button presses.

### Results

The number of button presses reported by the participants was identical to the number of button presses identified in the log entries for all participants. No further (i.e., accidental) button presses were present in the data.

### Discussion

While the number of participants in this study was small, the range of situations in which the wearables were worn was likely sufficient to cause accidental presses (i.e., during the everyday life of participants). For sleeping periods, we can rule out the possibility of conscious avoidance of pressing the button. If buttons were overly sensitive, involuntary movement during sleep would have triggered a button press. We cannot fully rule out the possibility that participants had a heightened attention to not pressing the button while awake. This explicit instruction could not be avoided, because without any instruction participants might accidentally or intentionally press the button without making note of the event, making it difficult to measure unintentional presses. This setting is also comparable to a regular study setting, where participants have the instruction to only press the button under certain conditions and avoid any further inputs. Considering this, we expect that participants within a study setting would be able to avoid erroneous or accidental inputs.

This raises the question of whether or not the device registers all intentional presses. The device’s feedback options (i.e., vibration and light signal) should ensure that participants are able to recognize when the wearable has registered an input. Yet, to test the possibility that intended button presses by participants are not registered by the wearable (e.g., button not firmly pressed, data storage erroneous), we conducted a further empirical pilot study (pilot study 6).

While registering button presses reliably is useful for logging (e.g., the time of an occurring event), augmenting this information with accelerometer data allows the configuration application to use this data as a PAS, as described above. To assess the reliability of the PAS, we first assessed the reliability of angle measurements made without human estimation. Pilot study 4 assesses the device’s influence itself (e.g., position of the board within the wearable’s casing) on angle measurements by using fixed angles without the devices’ wrist bands. Pilot study 5 expands on this with the use of wrist bands and the measurement of multiple different angles.

## Pilot study 4: Test of synthetic angle measurements without wrist bands

To be comparable to the VAS, the PAS needs to be accurate across several domains. We discuss the accuracy of estimation by humans using the PAS in more detail in pilot study 6. The angle measurement itself must also be reliable; that is, having the device at a fixed angle must produce the same logged value. While datasheets indicating the accuracy of the device accelerometers are available, this device-related reliability is also influenced by other factors. Sensor-specific accuracy would be sufficient for purely relative continuous real-time measurements (e.g., to track movement). However, in the specific application of the PAS, the absolute orientation of the sensor is relevant and may be affected by the relative orientation of components to each other. Each of these components might be influenced by deformations due to the action of pressing a button to obtain a measurement (i.e., the position of the sensor board within the plastic casing, the plastic casing in the rubber wrist band, and the rubber wrist band on the wrist). Furthermore, the anatomy of the wrist is neither uniform nor rigid, introducing another factor that can influence reliability. To assess these influences, we obtained measurements from wearables without wrist bands, affixed to a flat surface.

### Method

#### Materials and procedure

Four wearables were used in this test. The wearables were used without the elastic wristband and were affixed to a table using adhesive tape. All wearables were configured to log acceleration on a button press, as well as activate their LED and vibration motor. The latter two configurations will likely be used as feedback for participants in a real application, and, hence, were set to ensure that these settings have no influence on the measurement. Each wearable button was pressed ten times in quick succession.

### Results

We analyzed the measured device angles on the *y*-axis because this is the axis of interest for the PAS. All devices were affixed flat to the table; as such, they were expected to measure an angle close to 0°. We did not expect exactly 0° because the table was not ensured to be exactly level, and the circuit board orientation within the plastic casing might result in an angle.

Table 3 shows a summary of data from the four wearables. A Kruskal–Wallis *H*-test indicated significant difference across wearables, *H* = 33.40, *df* = 3, *p* < .001. The devices also significantly differed in the variability of measured angles according to a Levene test, *F*(3, 36) = 4.98, *p* = .005.

**Table 3:** Summary of angle data for each wearable from pilot study 4

Wearable	*M*	*SD*	Min	Max	Range
1	0.12	0.32	– 0.65	0.37	1.02
2	– 4.55	0.27	– 4.91	– 4.02	0.89
3	– 2.53	0.84	– 3.68	– 0.94	2.74
4	– 1.82	0.96	– 3.11	– 0.84	2.26

### Discussion

While the wearables significantly differed in the measured mean angle, this difference did not exceed 5°. This is likely due to slight differences of circuit board orientation within the casing. Furthermore, the range of measurements did not exceed 3° on any wearable, suggesting that measurements are generally consistent within wearables. Therefore, while slight differences were observed, these are minimal and should not introduce a substantial bias to measured angles, especially compared to the expected inaccuracy of a human user estimating an angle.

## Pilot study 5: Test of synthetic angle measurements with wrist bands

After assessing the precision of the wearables’ acceleration-based angle measurement, we also investigated the role of the rubber wrist band in the accuracy of wearables, including their angles. The rubber band might slightly yield when the button on the wearable is pressed; therefore, the orientation change of the device caused by the button press might be amplified.

### Method

**Fig. 7:**
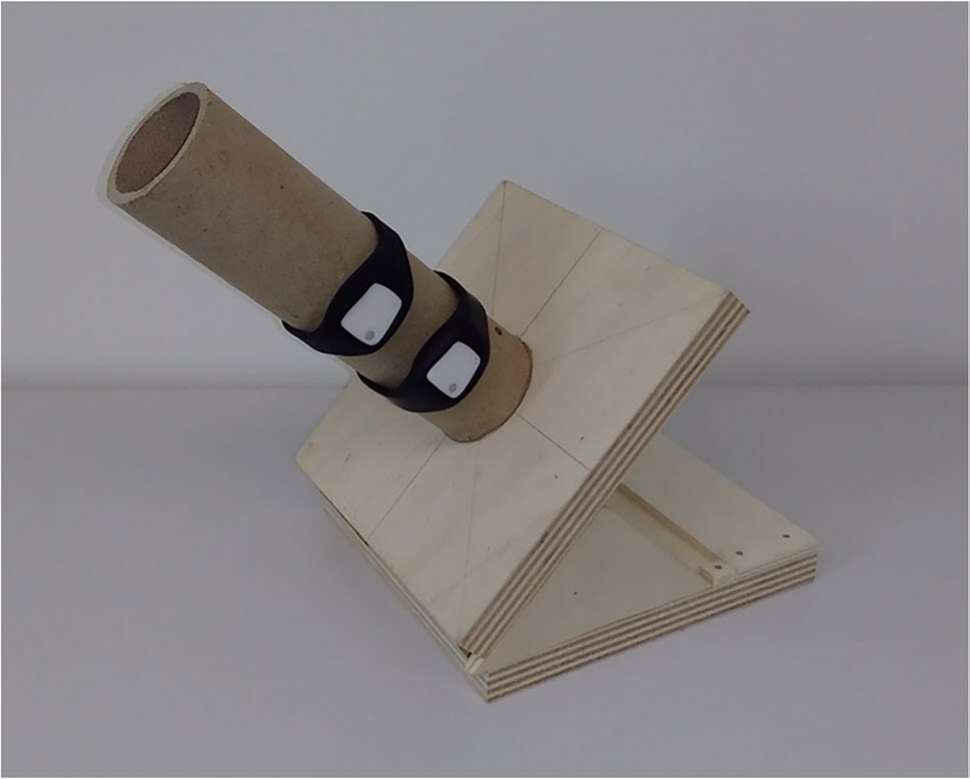
The device used to fix wearables to specific angles

In order to set the wearables to specific angles, we constructed a device consisting of a thick cardboard pipe fixed to a wooden plate on a hinge. Figure 7 shows this device. In its neutral position, the pipe is vertical (i.e., 90° from the ground plane). A wooden cutout triangle enables reliably aligning the pipe diagonally (i.e., 45° to the ground plane) or horizontally (i.e., 0° to the ground plane).

Four wearables were strapped to the pipe using the rubber wristbands that would also be used by participants to wear the device. Each wearable was configured to log the acceleration on a button press. The pipe was then set to three different angles (0°, 45°, 90°). Each wearable was pressed nine times per angle. The pipe was rotated between each measurement, changing the orientation of the device’s *x*- and *z*-axes, but preserving the orientation of the measured *y*-axis.

### Results

Table 4 shows data from each wearable.

**Table 4:** Summary of measurements for each wearable and angle from pilot study 5

	Target angle								
	0°			45°			90°		
Wearable No.	*M*	*SD*	Range	*M*	*SD*	Range	*M*	*SD*	Range
1	– 1.25	4.09	9.71	45.19	6.94	22.21	78.69	1.65	4.85
2	1.95	1.23	4.08	46.67	2.39	8.69	89.57	1.11	3.50
3	– 0.84	4.82	12.09	44.26	3.25	9.88	85.52	1.90	6.22
4	2.11	1.49	4.34	43.75	4.62	14.74	86.72	1.71	5.87

All values are in degrees

### Discussion

Data suggest that the target angle can be recovered relatively closely on average, with some device-dependent variations. Wearable 1, for example, shows both the highest range of measured values in the 45° condition, and the highest deviation from the target value in the 90° condition. Such effects might be due to a loose wrist band, a problem that is also likely to occur in real use of the device. Overall, the values vary the most in the 45° condition. Considering Eq. (1), one can see that small deviations in acceleration should impact angles close to 45° to the greatest extent, which fits the observed findings. Thus, the deviations observed in this condition should be representative of the maximum expected deviations, considering the use of a rubber wrist band and pressing of the button for a measurement.

## Pilot study 6: Validation of angles, Likert scale, and PAS

In the previous pilot studies, we investigated the basic properties of the wearable itself. The next steps are to consider the input options available when the wearable is in use. Utilizing only the button allows measurements on a Likert scale by counting the number of presses. Utilizing the accelerometer allows measurements on a PAS.

Both measurement options come with challenges compared to their pen and paper (or digital) counterparts. With a visual Likert scale, all possible values coexist, and the appropriate value can be selected (and usually modified); however, with the button input, the value is represented by a sequence, dependent on the users’ ability to keep the current count in memory. To aid the counting procedure, the wearable has a function to change the LED color with each button press, giving the users better feedback on when a button press has been registered. However, there are two other issues with the counting procedure: first, erroneous additional inputs cannot be corrected by the users; second, long delays of > 3 s between inputs will result in two separate measurements in the data file.

On the PAS, on the other hand, all possible values coexist. However, compared to the VAS, the PAS has its own issues. First, the PAS is highly dependent on the users’ ability to estimate angles. While users are able to judge specific points on a VAS fairly accurately (Reips & Funke, 2008), the same might not be true for the PAS. This is further compounded by the deformations that can occur when the button is pressed, as mentioned above in pilot study 4. Second, while users can easily set the value of the VAS to its end points, the end points of the PAS might actually be relatively inaccurate. As the position and orientation of the device on the user’s wrist cannot be predicted, the available data can effectively only be used to determine the polar angle in a spherical coordinate system. Therefore, angles above 90° (i.e., beyond the vertical apex point) result in mirrored values. This means that, while very intentional lower end measurements (i.e., angles noticeably below the horizontal) might be accurately identified, the same intention on the upper end (i.e., angles noticeably beyond the vertical) would result in attenuated values.

To validate the use of these scales on our wearable, we performed another pilot study using a within-subject design, by comparing values from Likert-type scales and PAS obtained from wearables to their counterparts on smartphones (visual Likert-type scale and VAS). Beyond that, we also tested participants’ ability to estimate predefined angles (0°, 45°, 90°), replicating the design from pilot study 5.

### Method

#### Participants

A total of *N* = 58 participants took part in this study. Participants were students at the Karl Landsteiner University of Health Sciences. The majority were female (*N*_*female*_ = 52, *N*_*male*_ = 5, *N*_*other*_ = 1). Participants had a mean age of *M* = 22.24 years (*SD* = 3.88, range = 18-37).

#### Materials

Each participant was provided with a wearable. The wearables were programmed to log the number of button presses as well as the angle during each button press. For comparison, participants used their personal smartphones to fill out questionnaires in the web-app-based ESM software ESMira (Lewetz & Stieger, 2023).

The eight extraversion items of the German version of the Big Five Inventory (BFI) were used (Lang et al., 2001; Rammstedt, 1997). These items were answered both on an analog scale (VAS or PAS, depending on input device) and a five-point Likert scale (visually on a smartphone, or via wearables by pressing the button for 1–5 times). The BFI was selected because it usually has means near the center of the scale, and some spread across the scale (John and Srivastava, 1999; Lang et al., 2001). Participants were, therefore, likely to vary interindividually, thus making full use of the scale overall. Furthermore, BFI is a trait concept that should be stable during the assessment phase across devices.

#### Procedure

Participants were first provided with the wearable and allowed to familiarize themselves with it. They were then instructed on how to access the questionnaires on the ESMira platform and confirmed the functionality of ESMira by filling out an initial questionnaire about demographic data and their wearable number for matching purposes.

First, participants were instructed to set their lower arm to 0° (i.e., horizontal position), then 45° (i.e., diagonal position), and finally 90° (i.e., vertical position). Participants pressed the button once for each angle to make a measurement. Second, participants answered each of the eight extraversion items first by using the VAS on the smartphone, and then the PAS on the wearable, before moving on to the next item. Third, participants again estimated angles, this time in descending order from 90°, 45°, down to 0°. Fourth, participants answered the extraversion items again, but on a five-point Likert-type scale: first, on the smartphone using the classical visual representation of the Likert scale; second, on the wearable by pressing the button from 1 to 5 times depending on the desired value.

#### Analysis

During coding, wearable data were assigned to one of the conditions (i.e., first or second angle measurement, Likert scale measurement, or PAS measurement). Due to some erroneous entries, some data could not be assigned (e.g., if there were nine instead of eight measurements in the PAS condition). Data that could not be clearly associated with a condition were discarded.

### Results

#### Angle data

**Fig. 8:**
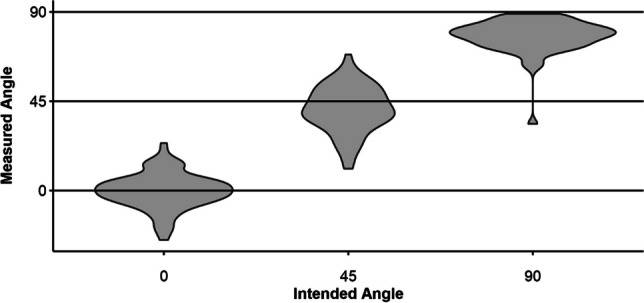
Violin plots of measured angles for each target angle from pilot study 6

After coding, data from all 58 participants were available. However, six individual angle blocks (5.17%) were discarded, leaving a total of 330 observations (110 per angle). The mean angles were 0.25 (*SD* = 8.91) in the 0° condition, 40.89 (*SD* = 11.66) in the 45° condition, and 77.60 (*SD* = 8.59) in the 90° condition. Figure 8 shows the corresponding violin plots.

**Fig. 9:**
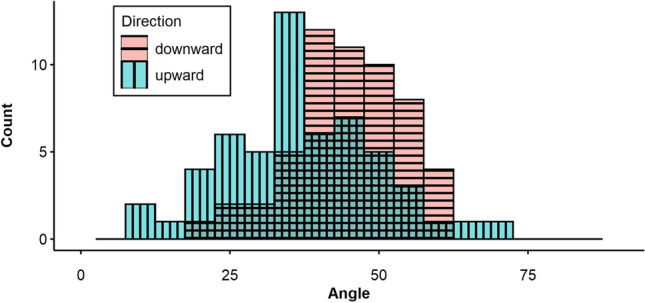
Histograms for the 45° target angle from pilot study 6, separated for upwards and downwards estimation

Test–retest reliability was also assessed for the 45° angle specifically. This target angle was of interest because it was preceded by another angle in both instances (i.e., it followed the 0° target angle in the first angle estimation sequence, or the 90° target angle in the second). Thus, its estimation was approached from different directions for each measurement. After discarding six incomplete pairs (10.34%), data from 52 participants were left. Figure 9 shows histograms of the estimated angles for each of these two measurements. The right-shifted distribution in the downward-condition compared to the upward-condition indicates that participants’ estimations of the 45° target angle were higher in the latter. A Wilcoxon signed-rank test indicated that the distribution for the upward estimation (*M* = 37.13, *SD* = 12.97) and the downward estimation (*M* = 44.52, *SD* = 9.27) significantly differed in their location (*d* = 0.66, *V* = 297, *p* < .001).

#### Likert scale data

After discarding one Likert scale data block (1.72%), data from 456 individual observations across 57 participants were available for the Likert scale comparison. Table 5 shows the correspondence between the wearable and the smartphone devices.

**Table 5:** Comparison of Likert scale entries per device from pilot study 6

		Smartphone				
		1	2	3	4	5
Wearable	1	61	0	0	0	2
	2	0	103	1	0	1
	3	0	0	86	2	0
	4	0	0	1	137	2
	5	0	1	0	1	58

The Spearman rank correlation between smartphone data and wearable data on the Likert scale was *r* = .96 (*p* < .001). A total of 11 observations (2.4%; generated by five participants) did not match up; however, a χ^2^-test did not indicate that this deviation was significant (χ^2^ = 0.11, *df* = 4, *p* > .999). Cohen’s κ also indicated high agreement between the scales (κ = .97; 95% CI: .95, .99).

#### Analogue scales data (i.e., VAS and PAS)

After discarding three PAS data blocks (5.17%), data from 440 observations across 55 participants were available. The angles of the PAS were rescaled for better comparability between scales, so that the maximum angle (i.e., vertical scale end) would be at a value of 100 instead of 90. Two data points were identified as outliers because the difference between scales exceeded 50 (i.e., more than half the scale) and hence were removed, leaving a total of 438 observations for analysis.

On the item level, the two scales (PAS vs. VAS) were highly correlated over all items, *r* = .89 (*p* < .001). On the scale level (i.e., with the items averaged calculating the Extraversion score), the correlation increased to *r* = .95 (*p* < .001). These high correlations, as well as inspections of scatter plots, indicated a linear relationship. A linear mixed effects model was used to further assess correlations (see the online supplement for the model specification) with a multilevel approach to account for the grouping of data points within participants. The regression was performed on transformed data, with the difference between PAS and VAS serving as criterion variable, and the VAS values as predictor variable. If both scales were equal, this difference should be 0 across all possible VAS values, resulting in a slope of zero.

**Fig. 10:**
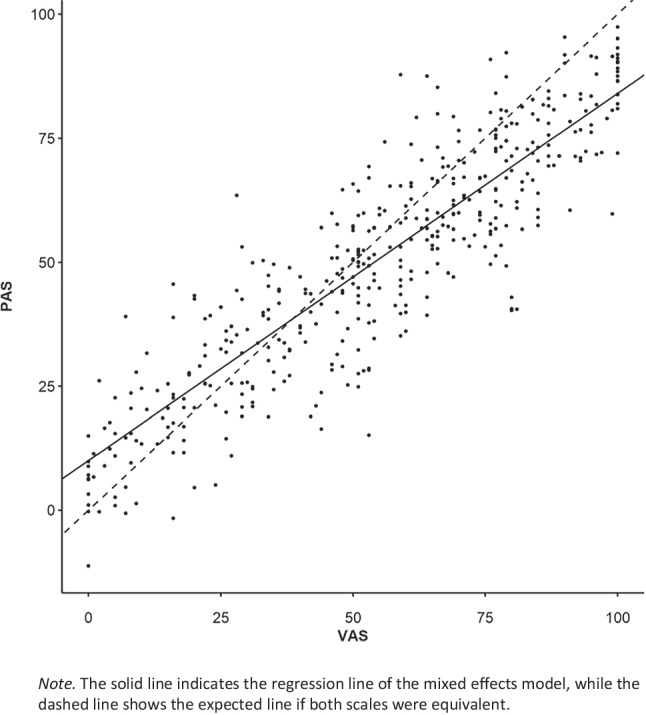
Scatterplot of VAS values and corresponding PAS values from pilot study 6

The results of this model are shown in Table 6. The VAS value predictor had a significant slope, indicating a measurable bias. The positive intercept of 9.34 indicates that the PAS significantly overshoots the VAS at the lower scale end (i.e., for VAS values close to 0). The significant negative slope of – 0.25 indicates that PAS values do not grow as fast as VAS values, resulting in the PAS values being lower than corresponding VAS values at the other scale end (i.e., values close to the VAS scale end at 100). This is also evident by the mismatch between the regression line and the expected line in Fig. 10.

**Table 6:** Results of linear mixed effects model for analogue scales data from pilot study 6

	Fixed					Random
	*B*	*CI*	*SE*	*t*	*p*	*SD*
Intercept	10.03	6.78; 13.28	1.65	6.07	< .001	10.26
VAS value	– 0.26	– 0.30; – 0.22	0.02	– 11.68	< .001	0.12

*N*_*VPN*_ = 55, *N*_*obs*_ = 438, ICC = .26

ICC = intra-class correlation of the null model

### Discussion

Considering the angle data overall, a similar pattern of deviation as in pilot study 5 was observed, such that there were lower deviations at the scale ends compared to the middle. The standard deviations were considerably higher than that in pilot studies 4 and 5; however, this was expected because this condition adds further variance to the orientation of the device (e.g., the wearable on the wrist might be at a slight angle to the overall angle of the lower arm, and the wrist band might deform more if worn loosely). The means of the angle estimations suggest a progressive negative bias, with 0° being the most accurately estimated target angle, and 90° undershooting the most. These observations are similar to those in pilot study 5.

The angle estimation also seems to be influenced by the direction of the measurement; for example, whether a participant raises (vs. lowers) their arm to reach the target angle. Participants typically *raise* their arm for a measurement in most situations. Therefore, we expect that such an effect would have little influence in a typical study of this kind.

The comparison of PAS and VAS shows a pattern similar to that of the angle estimations on the upper scale end, with participants increasingly underestimating values on the upper end of the VAS. However, in contrast to the direct angle estimation, in which the 0° angle was the most accurately estimated angle, the PAS shows an initial overestimation of low values on the VAS. Overall, this creates a pattern of PAS values being attenuated towards the center of the scale, compared to the VAS. Despite these mismatches, the scales remain highly correlated, particularly when averaged. This suggests that the two scales are similar in their properties. Comparisons across the scales would be difficult to correctly interpret; however, comparisons within the PAS should appropriately discriminate values.

In contrast to the PAS-VAS pairing, the Likert scale does seem to be directly comparable. The data show only occasional mistakes with the Likert scale, with most measurements (97.6%) matching up between both input methods. This suggests that participants are generally capable of counting and entering correct values.

One limitation of the PAS is that it may be influenced by gender. Male participants are typically better at spatial ability tasks (e.g., mental rotation, Maeda & Yoon, 2013) than female participants. This might influence the estimation of angles. Effects of gender were not assessed in the present study, due to only five male participants in the sample. See the supplement for a descriptive comparison of males and females.

## Main study: Event compliance in a field setting

The final study investigated the effects of the wearable device on participants’ event compliance; that is, reacting to certain events in daily life and logging them using the device. Event-based scheduling places responsibility on the participants because they must react to a signal reminding them of their study participation and also actively build an association between an event in their lives and filling out a questionnaire about this event.

In this study, laughter was chosen as an ‘event’ because it is a potentially frequent, transient, and overt behavior. Laughter has been studied previously using diary methods. For example, Kambouropoulou (1930) and Graeven & Morris (1975) had participants record detailed descriptions of laughter events. Mannell and McMahon (1982) and Martin and Kuiper (1999) also had participants record laughter events using short pen-and-paper questionnaires, akin to using event-contingent scheduling in ESM designs.

One of the first studies using a one-button wearable for self-tracking events was a case study by Larsen et al. (2017). To date, there has been no investigation of the effects that one-button wearables have on compliance. Participant burden is an important driver of motivation, and, thus, compliance. We hypothesized that, when used properly, one-button wearables would lower the burden for participants compared to smartphones. Compliance with recording events is hard to assess because a validated reference value of event frequency is not available. However, given random assignments to groups, we can assume that the average frequency would be equal across groups. Further, considering that participants are far more likely to not report an event rather than report events that have not happened, either intentionally or by accident (see pilot study 3), we can assume that burden would bias the results by reducing the count of logged events. We, therefore, assume that an observed difference in the count of logged events indicates that the group with the higher count is closer to the actual number of events (i.e., a higher fraction of events that happened have actually been logged). Thus, while actual event compliance remains unknown, a difference in logged event count can be interpreted as the group with higher logged event count having better event-compliance. Hypothesis 1 is, therefore, that the group using one-button wearables will have a significantly higher number of events logged on average than the group using smartphones in an experimental design with random assignment to groups.

Due to the lower burden, we also expect the participants in the wearable group to be less likely to delay entering an event. We would, therefore, expect to see a bias for later event times in the smartphone group. Therefore, Hypothesis 2 is that the average time of day for logging laughter events is earlier in the wearable group compared to the smartphone group.

Lastly, as we measure happiness, both on a VAS and a PAS, depending on the device used, we can compare the two measuring methods, similarly to pilot study 6. At the time of planning of this study, we expected both measurement methods to work similarly; thus, our Hypothesis 3 is that the mean happiness scores are equal across the one-button wearable and smartphone groups.

### Method

#### Preregistration

The design plan, sampling plan, analysis plan, and hypotheses of this study were preregistered on the Open Science Framework (OSF) and can be accessed at https://osf.io/yjgfu. However, there were some deviations from the preregistration. First, we did not reach the preregistered sample size. See the participants section below for a rationale on why we believe the used sample size is sufficient for the current study. Second, we stated in the preregistration that we would exclude participants who did not own an Android smartphone. However, we had since acquired Android smartphones to lend to participants, which allowed us to also include those who did not own an Android smartphone. Furthermore, the application became available on iOS later in the data collection phase, allowing for the inclusion of participants with iPhones. Third, we expanded the data cleaning procedure and revised one preregistered exclusion criterion; these changes are further explained in the statistical analysis section.

We deviated from the analysis plan for Hypothesis 1. Initially, we mentioned the use of a *t*-test in the preregistration for this analysis. However, we realized later that generalized mixed effects models (specifically negative binomial mixed effects models) are more appropriate for count data. Furthermore, we realized over the course of data collection that we had several other variables that could serve as indicators for participant motivation. We, therefore, include these as covariates in our model for Hypothesis 1. We made sure that these changes would not lead to different main findings by including the originally preregistered analyses in the supplement. Beyond the preregistered analysis, we performed some exploratory analyses, which were not preregistered, and which are reported in their own section below.

#### Participants

A power analysis, using the smallest effect sizes reported by Martin and Kuiper (1999), *r* = .1, was used as benchmark for expected comparable effects of personality on laughter. Using this effect size, a power calculation indicated a required sample size of *N* = 614 (α = 5%, power = 80%, one-sided). However, the longitudinal design increases power due to the repeated measurements. Using an approach presented by Twisk (2006, p. 123), we calculated a required sample size based on the expectation that we would obtain at least one observation per day from each participant (thus using 28 as number of level 1 units), and an expected intraclass correlation coefficient (ICC) = .3, resulting in the preregistered sample size of *N* = 200. However, this sample size was based on the smallest expected effects for personality. The main group difference (i.e., Hypothesis 1) was expected to have a small-to-medium effect size (*r* = .2) which, as mentioned above, would result in a total sample size of *N* = 50. Therefore, we believe the reached sample size of 134 to be sufficient for this study.

A total of 167 participants began participation in the study (82 in the wearable group, 85 in the smartphone group). Of these, 147 participants finished the study by completing the final questionnaire (70 in the wearable group, 77 in the smartphone group). Thirteen participants (11 from the wearable group, two from the smartphone group) were excluded due to technical issues (e.g., malfunctioning devices, loss of data) or not completely following the study protocol (e.g., indicating that they only participated on weekends). The analytic sample size was, therefore, *N* = 134. Of these, 59 participants were assigned to the wearable group and 75 to the smartphone group. The difference in group size was due to a larger dropout rate in the wearable group. However, dropout was not statistically significant according to a binomial test (*p* = .195), and the groups were similar in their demographics. Participants in the wearable group were mostly female (66%; 39 female, 20 male) and, on average, aged 30.47 years (range, 18–74, *SD* = 14.22). Participants in the smartphone group were also mostly female (68%; 51 female, 23 male, one other) and, on average, aged 31.11 years (range, 18–85, *SD* = 13.79). Overall, participants were mainly female (67%; 90 female, 43 male, one other) and, on average, aged 30.83 years (range, 18–85 years, *SD* = 13.93).

#### Design and procedure

Each participant was invited to an introductory meeting. Participants were first given an overview of the study, and then signed an informed consent form if they decided to participate. During this meeting, participants were assigned to one of the experimental groups (wearable vs. smartphone) according to a pre-randomized list in order of appearance. Both groups used their smartphones with the application ESMira (Lewetz & Stieger, 2023). Due to ESMira not being available on iOS (i.e., iPhones) for most of the data collection period, 17 participants (12.7%) who were iPhone users were given Android smartphones with ESMira preinstalled to borrow. As mentioned above, this was a deviation from the preregistration. Participants were then given the required equipment (i.e., a wearable in the wearable group, or a borrowed smartphone if necessary) and were assisted in setting up ESMira. After this initial meeting, participants were asked to follow the study protocol for 28 days.

Laughter events were defined as a ‘belly laugh’ or ‘fit of laughter’. A belly laugh was defined as follows: “A belly laugh means a sincere, loud laughter, coming from the heart. It moves the whole body, especially the belly and chest. It is characterized by rhythmic movement of the diaphragm and is usually triggered automatically.” A fit of laughter was defined as follows: “A fit of laughter is a severe laugh lasting a longer time, accompanied by tears and the feeling of being unable to stop.” The exact German definitions given to the participants can be found in the supplement.

The general task for the daily routine was to log laughter events, but only if it met the definition of a belly laugh or a fit of laughter (exact definitions were given during the introductory meeting). Whenever a laughter event occurred, participants indicated this on their device, and also indicated the type of laughter (i.e., belly laugh or fit of laughter) and rated their happiness during the event. In the wearable group, laughter type was indicated by pressing the button either once or twice (for belly laughs or fits of laughter, respectively). Happiness was rated using the PAS. In the smartphone group, the type of laughter was a selectable item (technically realized as a two-point Likert scale), and happiness was rated via a VAS.

In addition to this event-based measurement, participants would also receive three pseudo-random bings per day (between 9:00 a.m. and 6:00 p.m.), prompting them to rate their current happiness independent of any laughter. In the wearable group, the bing would be signaled by the device vibrating and activating its light, to which participants were asked to react with a PAS measurement (this measurement was marked as a reaction by using three button presses). In the smartphone group, the bings were provided as notifications, unlocking a single-item questionnaire with a VAS.

Participants in the wearable group were instructed to consistently wear the device on the same arm and in the same orientation, given the findings from our pilot study of the PAS mentioned above. Most participants reported adhering to this instruction, with only one participant indicating in the cross-sectional questionnaire to have switched sides at least once. Almost all participants also reported that they made sure the orientation was consistent, with only four participants reporting that the orientation was changed at least once^1^.

At the end of the day, following a customizable bing, which was defaulted to occur at 8:00 p.m., participants in all groups filled out a short end-of-day questionnaire on their smartphones using the ESMira application.

After 4 weeks, participants completed a final cross-sectional questionnaire on an online platform, which included items to assess demographics and personality. Participants were thanked and debriefed and offered an overview of their personal data if they were interested.

#### Materials

The daily end-of-day questionnaire consisted of four questions. For the first two questions, participants were asked to recall or estimate how many laughter events of each category they believe they had forgotten to log. Participants were also asked to provide the time they went to bed the previous night, as well as the time they got out of bed in the morning. These last two items were used to group laughter events according to waking periods rather than strictly by calendar dates (i.e., if a participant stayed up beyond midnight and experienced a laughter event, this event could be associated with the date of the previous day).

The final, cross-sectional questionnaire included some custom questions, as well as multiple established scales. For sociodemographic data, we asked for age and gender. Further concepts were assessed which were not analyzed as part of this study (i.e., satisfaction with life, personality, gelotophobia, cheerfulness).

#### Statistical analysis

Some participants continued the study protocol for several days after they were due to terminate it. We chose to exclude any data after the 30th day. This allowed us to use some of the additional data while keeping the risk of including erroneous entries low. There were instances of multiple responses to bings in both groups. In the wearable group, this was mainly caused by participants pressing the button multiple times, while in the smartphone group, there was an occasional software bug that allowed participants to fill out the questionnaire for a single bing multiple times. We chose to only keep the first response within 30 min after each bing, removing 13 entries (0.3%). This was done for compatibility between groups, as the questionnaires were only available for 30 min after each bing in the smartphone group. Event entries were then summed on each day for each participant. If a participant had not reported any events, but other data were available (i.e., responses to signals or the end-of-day questionnaire), the count was set to zero. However, if no data at all were available from a participant on a particular day, that day was removed. This resulted in 495 (13.2%) of all recorded days being removed. Note that the procedures described above were not part of the preregistration.

We originally overestimated adherence to the study protocol. In the preregistration, we stated that we would exclude subjects with no data on individual days (i.e., no event counts, no reaction to signals, and no filled out end-of-day questionnaire). Adherence to this protocol would, however, have reduced the number of analyzed participants to 52, thus drastically reducing power. Considering this, we changed our analysis to use mixed effects models, which will naturally give participants with sparser data less influence, and also analyzed the full data set. Furthermore, we included the number of missing days per participant as a predictor in our models, as this variable may also be an indicator of overall motivation.

We calculated random-intercept, random-slope, generalized linear mixed effects models to address Hypothesis 1. All analyses were done with the *GLMMadaptive* package in *R* (Rizopoulos, 2022). *GLMMadaptive* allows fitting generalized mixed effects models using adaptive Gaussian quadrature for a maximum likelihood estimation. The variances of the counts were considerably larger than the means in the descriptive data, indicating overdispersion comparative to a Poisson distribution, in which the variance and mean would be equal. The data were therefore assumed to follow a negative binomial distribution, which can model overdispersed count data. Overdispersion in the used parametrization of the negative-binomial distribution is modeled with the parameter φ. The relationship between mean and variance is σ^2^ = μ + μ^2^ / φ. Due to this inverse relationship, higher values of φ indicate less overdispersion (i.e., a variance close to the mean), while values close to zero indicate more overdispersion (i.e., high variance). Negative values would indicate underdispersion (i.e., a variance smaller than the mean). In the model, days (level 1) are nested within participants (level 2). All continuous level 2 predictors were grand-mean-centered. Furthermore, the *insight* package (Lüdecke et al., 2019) lacks functions to extract the residual variance from GLMMadaptive’s MixMod objects; therefore, we used the *lme4* package (Bates et al., 2015) to fit empty models to calculate ICCs. The final model is as follows:

- Level 1:
  $${\text{log}}\left(EventCoun{t}_{ti}\right)={\pi }_{0i}+{\pi }_{1i}Day+{\pi }_{2i}ComplianceDay+{e}_{ti}$$
- Level 2:
  $${\pi }_{0i}={\beta }_{00}+{\beta }_{01}ComplianceGeneral+ {\beta }_{02}MissedDays+{\beta }_{03}Wearable +{\beta }_{04}Wearable*Day+{r}_{0i}$$
-
  - Level 2:
- $${\pi }_{1i}= {\beta }_{10}+{r}_{1i}$$
-
  - Level 2:
- $${\pi }_{2i}= {\beta }_{20}+{r}_{2i}$$

To address Hypothesis 2, we calculated the average number of daytime laughter events per participant. Average time was measured in minutes since midnight. We also accounted for late events, coding events that occurred after midnight but before participants went to bed as belonging to the prior day (i.e., adding 1440 minutes). We then used a *t* test to test to assess the difference in means between the two groups.

Lastly, to assess Hypothesis 3, we performed a Mann–Whitney *U*-test for each measurement category (belly laughs, fits of laughter, reactions to bings) to test for differences in happiness between measurement methods (i.e., PAS in the wearable group and VAS in the smartphone group). We used a Bonferroni correction to account for multiple testing, resulting in an adjusted significance criterion of α = .0017.

### Results

Table 7 shows the descriptive data of counts. The wearable group had a mean count of total logged events over three times as large as that of the smartphone group. However, the (estimated) counts from the end-of-day data were much more consistent across groups. As expected, the frequency of fits of laughter was considerably lower than that of belly laughs.

**Table 7:** Daily means of event counts and end-of-day data from main study

	Event counts			End-of-day data		
	Belly laugh	Fit of laughter	Total	Belly laugh	Fit of laughter	Total
Wearable	4.65 (7.11)	0.56 (2.00)	5.21 (8.22)	2.56 (5.62)	0.47 (2.22)	3.03 (7.44)
Smartphone	1.53 (2.59)	0.16 (0.53)	1.69 (2.71)	2.87 (9.06)	0.21 (0.70)	3.09 (9.17)
Total	2.94 (5.37)	0.34 (1.42)	3.28 (6.13)	2.73 (7.70)	0.33 (1.58)	3.06 (8.43)

Standard deviations in parentheses

Initially, we intended to use the end-of-day data of forgotten events to supplement the logged events. However, after analyzing that data separately, we had concerns about the quality of those data (see supplement for detailed analysis). If those data were accurate, we would expect certain symmetries to the logged data; that is, because the overall event count is expected to be essentially invariant, effects in logged data should be mirrored in the estimates of forgotten events. For example, a higher logged event count in the wearable group should lead to a higher estimated count of forgotten events in the smartphone group, to compensate for that difference. These symmetries were lacking in the end-of-day data; as such, we decided against using these data in the main analysis.

**Fig. 11:**
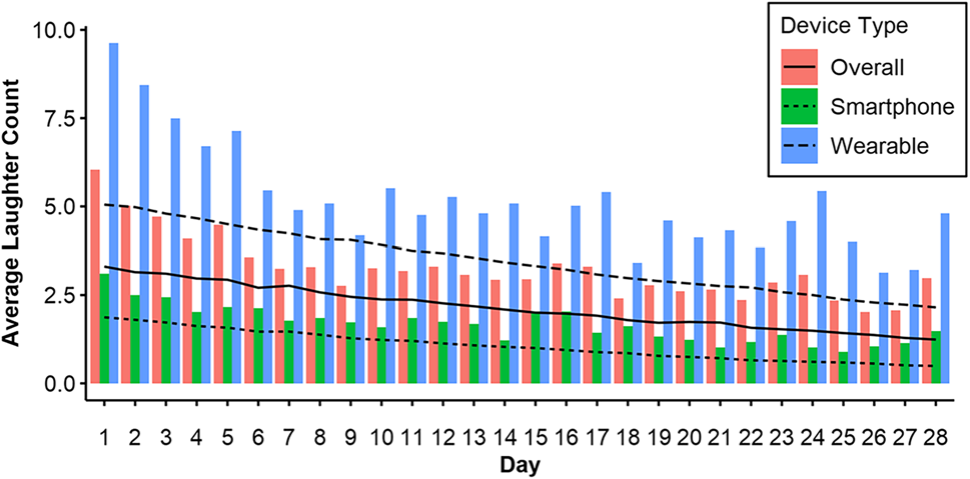
Measured and predicted mean event counts per day from main study, for wearables, smartphones, and overall average. *Note.* Bars indicate average measured counts; lines show mean-subject model predictions

Table 8 shows the results of the generalized mixed effects model described above. Of the analyzed predictors, all but the two signal-related compliance variables had significant effects on count. The log scale beta values are converted to multiplicative percentages in the following analyses. The expected difference between groups was significant, with counts 3.30 times higher in the wearable group, indicating support for Hypothesis 1. The day variable indicates a 5.18% decline in counts per day (i.e., 76.18% over the course of 28 days). The number of missing days also had a negative influence of 6.87% daily decline on the frequency for days with available data (e.g., a hypothetical participant with 28 missing days has an event count 85.36% lower than that of a participant with no missing days). Furthermore, the cross-level interaction between the group and day variable indicates an attenuated decline in the wearable group (3.21% decline per day, or only 58.61% over the course of 28 days, compared to the 76.18% in the smartphone group). The mean counts per day, as well as predicted counts per day, are shown in Fig. 11. See the online supplement for more detailed model diagnostics.

**Table 8:** Results of generalized linear mixed effects model for total laughter count from main study

	Fixed effects						Random effects	
	Coeff.	*B*	CI	*SE*	*z*	*p*	Coeff.	*SD*
Intercept	β_00_	0.203	– 0.244; 0.651	0.228	0.891	.373	*r*_0i_	0.915
Day	β_10_	– 0.053	– 0.065; – 0.041	0.006	– 8.746	< .001	*r*_1i_	0.032
Compliance Day	β_20_	0.135	– 0.037; 0.307	0.088	1.537	.124	*r*_2i_	0.370
Compliance General	β_01_	– 0.053	– 1.336; 0.212	0.395	– 1.424	.154		
Missed Days	β_02_	– 0.071	– 0.110; – 0.032	0.020	– 3.595	< .001		
Wearable	β_03_	1.195	0.847; 1.543	0.178	6.729	< .001		
Wearable * Day	β_04_	0.021	0.005; 0.036	0.008	2.557	.011		

*N*_*part*_= 134, *N*_*obs*_= 3,054, ICC = .58, *φ* = 1.61

Reference category for wearable was smartphone, ICC = Intra-Class Correlation of the null model

To test Hypothesis 2, a *t*-test between the average laughter time of day in the wearable group (*M* = 862.05 min, i.e., 2:22 p.m., *SD* = 215.35 min) and the smartphone group (*M* = 867.52 min, i.e., 2:27 p.m., *SD* = 231.02 min) indicated no significant difference (*t* = – 37.46, *df* = 123.48, *p* = .964; *d* = 0.01). Thus, we found no support for Hypothesis 2.

**Fig. 12:**
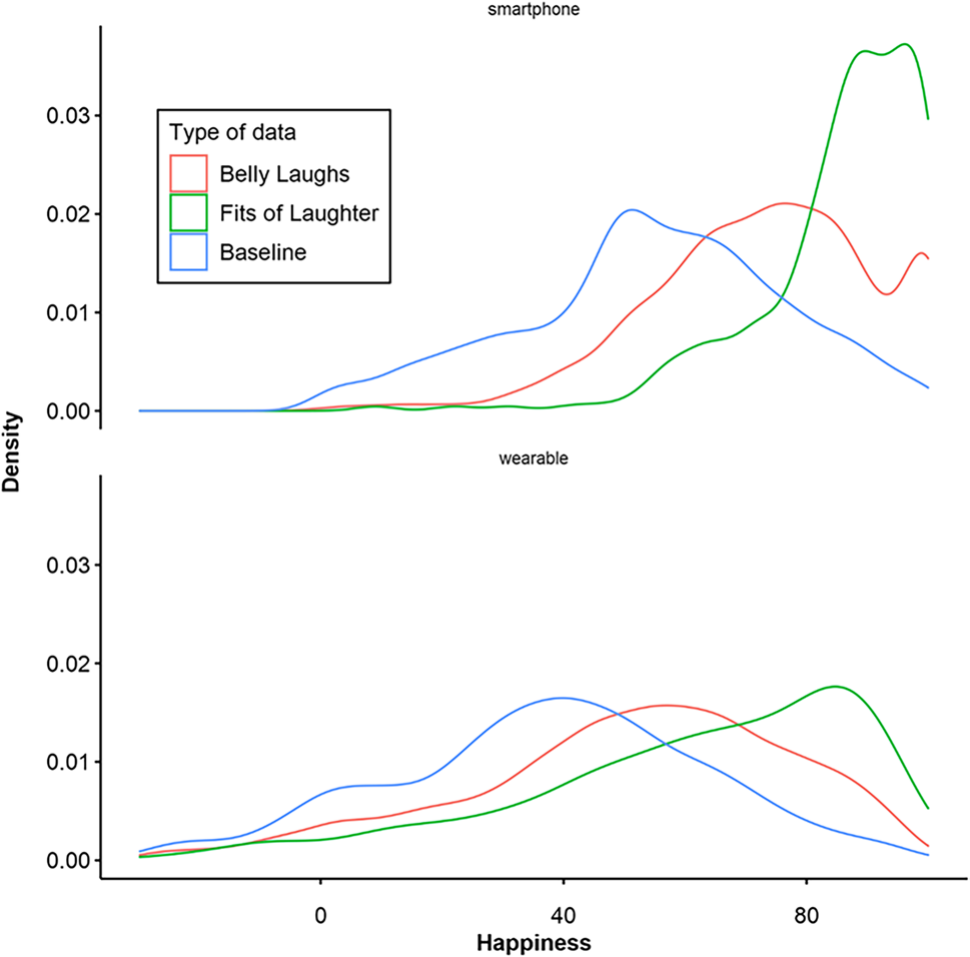
Density plots of happiness data from belly laughs (event-based), fits of laughter (event-based), and baseline happiness (signal-based bing reaction) from the main study

To test Hypothesis 3, concerning parity between the PAS and VAS, we compared happiness measurements provided on each of the device types (i.e., wearables and smartphones) in each of the three categories (i.e., belly laughs, fits of laughter, reactions to bings). There were some issues with happiness measurements in the wearable group. Seven (11.9%) participants in the wearable group indicated misuse of the PAS or similar problems in the debriefing (e.g., changing the orientation of the wearable as mentioned above or assuming the arm angle after pressing the button). Data from these participants were excluded from the following analysis. Of the remaining wearable data, 226 entries (1.6%) were removed as outliers, because they had values below – 30°. The resulting data consisted of 13,874 data points (8496 from the wearable group, 5378 from the smartphone group). Of these, there were 8184 from belly laughs (wearables: 5619, smartphones: 2565), 997 from fits of laughter (wearables: 725, smartphones: 272), and 4693 from bings (wearables: 2152, smartphones: 2541). As in pilot study 6, the PAS values were rescaled to have a maximum value of 100, to enable direct comparison between the measurement methods. Table 9 shows means and standard deviations for all categories, separated by group. Mann–Whitney *U*-tests indicated that the locations of the belly-laugh distributions (*d* = – 0.92, *U* = 10,833,807, *p* < .001), fit-of-laughter distributions (*d* = – 1.01, *U* = 159,230, *p* < .001), and bing-reaction distributions (*d* = – 0.73, *U* = 3,805,142, *p* < .001) all differed significantly between measurement methods. However, in both the descriptive statistics and visually in Fig. 12, the order of categories remained the same between measurement methods; that is, the bing reactions show the lowest, and fits of laughter the highest, happiness scores across both measurement methods, as expected. However, due to the change in location, Hypothesis 3 was not supported.

**Table 9:** Mean, standard deviation (in parentheses), and median of happiness for each type of measurement, separately for vas and pas measures from the main study

	VAS		PAS	
	Mean (SD)	Median	Mean (SD)	Median
Belly Laughs	72.87 (18.61)	74.00	50.48 (26.59)	53.58
Fits of Laughter	85.71 (13.92)	89.00	60.84 (27.59)	67.07
Bing-reaction (Baseline)	54.63 (21.91)	55.00	37.26 (25.57)	38.93

#### Exploratory analyses

As a further marker of burden, we compared signal-based compliance between groups (i.e., how often participants responded to the signal-based bings). A *t* test indicated no significant difference between the compliance in the wearable group (*M* = 0.49, *SD* = 0.29) and the smartphone group (*M* = 0.40, *SD* = 0.24) (*t* = – 1.89, *df* = 111.36, *d* = 0.34, *p* = .061).

Furthermore, we reanalyzed the data for hints of demographic influences, including gender and age as level 2 predictor variables. Due to the lack of available data, the one person with other gender was excluded from this analysis, resulting in a single binary gender variable. We also included whether participants were using a borrowed phone for the study, as well as the interaction of that variable with the used device. The following model was used:

- Level 1:
  $${\text{log}}\left(EventCoun{t}_{ti}\right)={\pi }_{0i}+{\pi }_{1i}Day+{\pi }_{2i}ComplianceDay+{e}_{ti}$$
- Level 2:
  $${\pi }_{0i}={\beta }_{00}+{\beta }_{01}{\mathrm {ComplianceGeneral}}+ {\beta }_{02}{\mathrm {MissedDays}}+{\beta }_{03}\mathrm{Wearable} +{\beta }_{04}\mathrm{Age}+{\beta }_{05}\mathrm{Gender}\;{({m})}+{\beta }_{06}\mathrm{BorrowedPhone}+{\beta }_{07}\mathrm{Wearable}*\mathrm{Day}+{\beta }_{08}\mathrm{Wearable}*\mathrm{BorrowedPhone}$$
-
  - Level 2:
- $${\pi }_{1i}= {\beta }_{10}+{r}_{1i}$$
-
  - Level 2:
- $${\pi }_{2i}= {\beta }_{20}+{r}_{2i}$$

Table 10 shows the results of this analysis. The effects observed in the main analysis remained significant. Neither gender nor age reached statistical significance. Participants with a borrowed phone logged significantly more events, but the interaction with the device type was not significant.

**Table 10:** Results of exploratory generalized linear mixed effects model for total logged laughter count including additional predictors from main study

	Fixed effects						Random effects	
	Coeff.	*B*	CI	*SE*	*z*	*p*	Coef.	*SD*
Intercept	β_00_	0.154	– 0.285; 0.593	0.224	0.688	.492	*r*_0i_	0.845
Day	β_10_	– 0.052	– 0.064; – 0.041	0.006	– 8.804	< .001	*r*_1i_	0.038
Compliance Day	β_20_	0.139	– 0.033; 0.312	0.088	1.583	.113	*r*_2i_	0.376
Compliance General	β_01_	– 0.430	– 1.169; 0.309	0.377	– 1.140	.254		
Missed Days	β_02_	– 0.074	– 0.111; – 0.037	0.019	– 3.915	< .001		
Wearable	β_03_	1.119	0.774; 1.464	0.176	6.361	< .001		
Age	β_04_	0.003	– 0.008; 0.013	0.005	0.498	.619		
Gender (m)	β_05_	– 0.068	– 0.383; 0.247	0.161	– 0.425	.671		
Borrowed Phone	β_06_	0.770	0.019; 1.520	0.383	2.010	.044		
Wearable * Day	β_07_	0.020	0.004; 0.035	0.008	2.495	.013		
Wearable * Borrowed Phone	β_08_	– 0.254	– 1.182; 0.674	0.474	– 0.537	.592		

*N*_*par*_= 133, *N*_*obs*_= 3,031, ICC = .58, *φ* = 1.60

### Discussion

In this study, we investigated how the use of wearable devices affects compliance in studies using event-based scheduling. We found significant evidence of some motivational effects, with more complete datasets leading to higher counts, as well as a decline in event counts with ongoing study duration. Furthermore, we found the expected group difference in the event count, with wearables resulting in a count of three times as high as that of smartphones. An interaction between the device and study duration also suggests that the motivational decline over time is attenuated in the wearable group.

We had a larger sample in the smartphone group than the wearable group. The drop-out rate was slightly larger in the wearable group, with 12 participants from the wearable group dropping out before completion, while only eight participants from the smartphone group dropped out. A bigger difference between devices was due to technical difficulties (e.g., corrupted data due to software bugs or malfunctioning wearables), which were subsequently solved throughout the ongoing study. However, the resulting sample did not statistically deviate from an even split.

In the exploratory model, the difference between participants using a borrowed smartphone for the study was significant. However, this additional influence did not notably change the effects found in the main analysis, nor did this variable interact with device type. Therefore, the use of borrowed phones and the inclusion of data from participants using them should not negatively impact our results. Rather, the effect of borrowed smartphones is positive. It might be assumed that an additional smartphone, particularly one that participants are not used to, would make participants less likely to use such a device and, therefore, log fewer events. However, it might be possible that the presence of an unfamiliar device served as a physical reminder for participants, more so than their regular smartphone.

Unfortunately, the required sample size calculated within the a priori power analysis was not met. However, to assess the reliability of the found effects, we calculated a post hoc sensitivity analysis. Using the approach by Twisk (2006), again with parameters from the study (i.e., 134 level 2 units, using the average of 23 observations per participant as the number of level one units, and the observed ICC of around .6), we calculated an effective sample size of ${N}_{eff}=179$. A sensitivity analysis in G*Power shows an effect size of *r* = .18 to be detectable reliably when testing two-sided with 80% power. The main effect of group assignment shows a correlation of *r* = .33 with the laughter count. This suggests that power might be suboptimal for some of the smaller effects found in this study, and that the main finding of wearables reaching higher event compliance than smartphones should be robust, lending support for Hypothesis 1.

Nonetheless, Hypothesis 2 could not be supported, suggesting that, while data from participants in the smartphone group is sparser, there is no bias to report events later. This may be influenced by our method, because the end-of-day questionnaire gave participants the option to account for events they might not want to log immediately, rather than delaying them and logging them later.

One noticeable disparity is the stark contrast in laughter frequencies reported in this study compared with previous research. Martin and Kuiper (1999) found an average of 17.6 laughter events per day, in line with Mannell and McMahon (1982), who reported an average of 13.4 overt laughter events per day. Lower frequencies were found in earlier studies by Graeven and Morris (1975) and Kambouropoulou (1930), who reported 6.1 and 6.0 laughter events per day, respectively. Our current findings (5.21 in the wearable group, 1.69 in the smartphone group) are closer to these latter results. A potential cause for the difference in our findings compared to the two more recent studies (Mannell & McMahon, 1982; Martin & Kuiper, 1999) is the operationalization of laughter. While those two studies included any overt laughter, the present study required a belly laugh, with the instruction specifically excluding “smaller” sorts of laughs like giggles. Thus, the present study’s laughter operationalization represents a subset of what was measured by previous studies.

Overall, the one-button wearable seems to have both strengths and weaknesses compared to the more generally applicable smartphone. The wearable performed well in the present design involving one input equating to two items. The lack of visual feedback means that differing prompts cannot be used, and, while multiple consequent inputs are technically possible, they require manual coding and are prone to error (see pilot study 6). The frequency of required inputs is most likely also important. If used for a more overt and infrequent type of event (e.g., eating a meal), then the relative burden of retrieving a smartphone might be smaller. Such events might also be less prone to recall errors, making alternative methods like the Day Reconstruction Method (Kahneman et al., 2004) viable.

A further limitation of one-button wearables is that the devices need to be handed out to participants in direct contact. Combined with the long study duration, this severely limited the number of potential participants. In this study, this was further complicated by the COVID-19 pandemic, which limited physical contact that could be made with participants.

We also had the opportunity to compare the PAS and VAS again in this study. Hypothesis 3 (mean happiness scores would be equal across groups) was not supported. However, this result is in line with the results of pilot study 6, despite the current study using a between-subjects design.

The variability was higher in the PAS data compared to the VAS data. Furthermore, the ordinal quality of the different categories is preserved between measurements, with both distribution means, medians, and the visually identified modes, showing the same distinct order in both measurement methods. Overall, this fits the assumption that both PAS and VAS behave similarly in their measurements but are not identical. Therefore, while comparisons *between* scales might be problematic, comparisons *within* scales should be feasible.

## General discussion

In this paper, we examined screenless, one-button wearables and their capabilities and feasibility for use in ESM designs. Larsen and colleagues (Larsen et al., 2017) have already demonstrated the viability of one-button wearables for self-tracking. Later, Stieger and colleagues (Stieger et al., 2020, 2022) further assessed the input options of one-button wearables with the introduction and use of the PAS. In the present publication, we introduce an open-source software that enables the use of one-button wearables, specifically the MetaMotionR by Mbientlab, in ESM designs. This software can handle the complete workflow, from configuring a device to downloading the data, within an easy-to-use and open-source Android application. Being open-source further opens this software up to the scientific community, making it easily available to users and open for further improvements and adaptations. Thus, this software makes the presented methods accessible to any interested researcher without requiring programming skills.

To judge the capabilities of the method, we investigated several basic properties of the used device, and furthermore tested it in realistic applications. Pilot studies 1 and 2 assessed the performance of the built-in battery. The measured battery runtimes were sufficient for the wearables to run continuously without charging for most required study durations. This was even the case for older, degraded batteries. Therefore, the battery can be viewed as an advantage of the one-button wearable device, because it reduces maintenance for the participant. Pilot study 3 showed the reliability of the push-button, by demonstrating that accidental, unintended button presses are exceedingly rare (i.e., none were observed in pilot study 3). This is important for ESM designs because participants generate data unobserved in the field. A reliable button ensures that the logged data are true entries. Furthermore, the results on the use of a Likert scale on the wearable in pilot study 6 also showed the button to be reliable for inputs, allowing participants to reliably enter an intended value (i.e., number of button presses). Therefore, a Likert (or categorical) scale appears to be a viable option for use with the wearable. Beyond the input method of press counting, the validity of the relatively novel PAS was of special interest. Pilot studies 4 and 5 assessed the one-button wearable’s basic measurement accuracy for acceleration-based angle measurements. Of these, pilot study 5 found results closer to the realistic baseline variability (i.e., before factoring in variability due to participants) and showed a standard deviation of around 7°. This increased to roughly 12° for freehand estimations by participants in pilot study 6.

We also investigated the PAS in more detail. Pilot study 6 compared the PAS and VAS within a person. This comparison showed a centering bias, whereby PAS values towards the ends of the scales were generally attenuated towards the scale center, compared to the corresponding VAS values. VAS and PAS were, therefore, highly correlated on the item level. The correlation further increased when comparing the two methods on the scale level (i.e., comparing the extraversion scores calculated with each measurement method). This suggests that the variability of the angle measurement, especially when adding variability of freehand angle estimation, might be randomly distributed, and thus would partially cancel out when multiple measurements are aggregated. The main study further compared the two methods in a between-subjects design. While there were clear differences in scale use between groups (e.g., the mean values were consistently lower for each category when measured with the PAS), certain patterns remained consistent. The order of categories was identical across measurement methods; for example, mean happiness measured by bing reactions was the lowest, mean happiness of fits of laughter was the highest, while mean happiness of belly laughs was between the two. This suggests that, despite numerical differences, both measurements captured the same construct across the two groups. Overall, the differences between the PAS and the VAS suggest that they are not directly equivalent. However, the high correlations between the two scales suggests that they are capable of measuring the same constructs. Therefore, the PAS poses a suitable option for an analog scale when using one-button wearables.

The main study mainly also investigated the effects that the use of one-button wearables had on event compliance. We found that over three times more laughter events were logged in the group using wearables than in the group using smartphones. True event frequency should be consistent across groups; therefore, we interpret this difference as a difference in the fraction of captured events. Despite the absolute fraction of events captured by each group being inaccessible, the measured difference is indicative of more missing events in the smartphone group. This is in line with previous research comparing ESM on smartphones and wearable form factors (i.e., smartwatches) that found increased compliance when wearables were used (e.g., Intille et al., 2016; Volsa et al., 2022). There was a clear difference in event-related compliance but only trend significance in signal-related compliance, most likely due to the low number of bings per day.

A further benefit of using wearables as input method is that they do not require the participant’s visual attention during a measurement. Button press counting is supported by a color-changing LED, and some participants might judge their arm angle visually when using the PAS. However, both inputs can also be used by exclusively relying on haptic and proprioceptive feedback. This can, theoretically, increase accessibility for participants with impaired sight or in situations with reduced visibility of the device. More importantly, this independence of visual attention could have been a contributing factor to the results of increased event compliance found in the main study. As previous findings suggest, laughter most likely occurs in social situations (Martin & Kuiper, 1999). However, while laughing with another person, it might not be possible, or socially acceptable, to interrupt the situation to interact with one’s smartphone. This could make participants in the smartphone group especially reluctant to enter an event. On the other hand, a social situation is barely interrupted when using one-button wearables, because access and use time are minimal, and participants could maintain eye contact when pressing a button during a social situation.

Despite the mentioned benefits, several limitations became apparent over the course of these studies. The most prominent were limitations of input and output. When the button press was used for a categorical input, as was the case in the main study, participants were required to remember the correct associations between categories and number of button presses. This is necessary as the only discernible output of the device is the colored LED, limiting the number of categories feasible for such an item. While in color changing mode, the LED cycles through three different colors, allowing for associations but requiring multiple associations with the same color if the number of categories exceeds 3. Using the button press for a Likert scale can also be problematic. While pilot study 6 showed that the use of a Likert scale is possible, the use of multiple consequential items can cause issues with coding. Moreover, because the device cannot handle questionnaires, manual assignment of responses to items may be necessary. In pilot study 6, some participants made an incorrect number of entries, invalidating that measurement occasion. These limitations apply less to devices with a screen (e.g., smartphones), which can display questionnaires. Furthermore, the wearable is limited in the number of signals it may elicit. While the number of bings may be sufficient for some designs it could be a limiting factor for others.

A further limitation shared with all specialized devices, compared to personal smartphones, is the requirement for physical interaction. ESM studies implemented via smartphone applications generally allow for remote administration, increasing the pool of viable study candidates. Wearables, on the other hand, must be provided to participants together with instructions on their use. A second meeting may also be required to return the device. Such commitments may reduce potential participants’ willingness to partake in a study using wearables.

The Main Study further exposed the wearables’ potential for error, resulting in missing, unusable, or corrupted data. Errors were far more pronounced in the wearable group than in the smartphone group, mostly due to initial points of failure (e.g., software bugs) and the lack of intuitiveness in using the device. We attempted to mitigate both of these problems by improving our protocol and instructions to participants, which partially helped. However, a key benefit of a smartphone, compared to a one-button wearable, is that participants are familiar with touch-screen user interfaces, and instructions can be displayed directly on a smartphone device. Another source of error is the necessity to consistently wear the device on the same arm and in the same orientation to use PAS. We specifically instructed participants to do so; however, some did not adhere to this instruction. A daily reminder or self-report check for wearing the device may, therefore, reduce errors.

Despite the limitations mentioned above, the main study showed that, when using a design appropriate for the device (i.e., two items per measurement), wearables can outperform smartphones in terms of reduced participant burden in handling the wearable. The access time on smartphones (e.g., retrieving and unlocking the smartphone, navigating to the appropriate application) is often disproportionately high compared to the usage time, especially in the context of questionnaires with only one or two items. Wearables, on the other hand, minimize access time because they are always ready for input. Therefore, wearables are more likely to satisfy the requirements of microinteractions (i.e., a total interaction time of 4 s or less) and are potentially less intrusive (Ashbrook, 2010). This is in line with Ponnada et al. (2017), reporting that four out of the first five participants in a smartphone group of a microinteraction-based ESM condition (i.e., 30 bings per day with single-item questionnaires) dropped out within the first few days, citing excessive burden.

It is important to consider how one-button wearables compare to other wearables, like smartwatches, which could allow dynamic presentation of information via a screen and provide more direct response options via their touchscreen. We suspect a tradeoff between these two options of wearables. Smartwatches might allow a user to request and input data with greater ease and would thus provide a good balance between the benefits of one-button wearables and smartphones. One-button wearables, on the other hand, are low maintenance because they need to be charged only infrequently, unlike smartwatches, which typically require charging daily or at least every few days. It is, therefore, likely easier for participants to keep one-button wearables in an operable state, reducing gaps in data collection due to empty batteries. It is also likely that one-button wearables require less attention for a measurement because, as mentioned above, one-button wearables can mostly be operated without looking at the device, relying on participants’ sense of proprioception. Inputting data on a device with a screen (e.g., smartwatch) might require more attention, which would reduce ease of use and make individual measurements less convenient for participants. Furthermore, not relying on a display for measurements might be beneficial for measurements in social situations, where interacting with a smartwatch screen could be perceived negatively by others, similar to phubbing. Ultimately, we believe that smartwatches should be another viable option for designs with frequent and short interactions. However, while one-button wearables restrict the scope of interactions by their limiting nature, we assume that designs using smartwatches need to minimize the scope of interactions as well. The small screen should be practical for quick glances and short inputs, but might be tedious for larger numbers of items or requirements of multiple precise inputs.

Overall, one-button wearables are a viable alternative to smartphones, provided the study design is suitable. One-button wearables involve single item microinteractions, with frequent measurements. When used for event tracking, the wearable is optimal for events that occur frequently (and thus accumulate high access times on other devices), in social situations, or that cannot easily be recalled later. Other devices, such as smartphones or smartwatches, may be more appropriate when the design necessitates longer questionnaires or dynamic elements such as varying questionnaires. Software options for smartwatches include systems introduced by Khanshan and colleagues (Khanshan et al., 2021) and Volsa and colleagues (Volsa et al., 2022). However, in situations where one-button wearables could be used, benefits such as increased data quantity, and probably also quality, are strong arguments to do so. Furthermore, with the provided open-source configuration application, one-button wearables are easy to use in research.

### Supplementary Information

Below is the link to the electronic supplementary material.

Supplementary file1 (DOCX 2194 KB)

## Data Availability

The datasets generated during and analyzed during the current study are available in the OSF repository, https://osf.io/6xhjn/. Pilot studies 1 through 6 were not preregistered. The main study was preregistered (https://osf.io/yjgfu).

## Footnotes

1 As Hypotheses I and II did not relate to the PAS, we included these participants in the corresponding analyses. Separate analyses excluding these participants did not substantially change the results.

## Code availability

The code used for analysis during the current study is available in the OSF repository, https://osf.io/6xhjn/.

The source code for the software used to configure the wearables during the current study is available in the following GitHub repository: https://github.com/KL-Psychological-Methodology/ESM-Board-Admin.
