# Supplementary material for: Development of an open-source solution to facilitate the use of one-button wearables in experience sampling designs
Source: Behav Res Methods. 2024 Jan 17;56(6):5876–99. doi: 10.3758/s13428-023-02322-y (PMC11335931; doi:10.3758/s13428-023-02322-y)

Supplement for: Development of an Open-Source Solution to Facilitate the Use of One-Button Wearables in Experience Sampling Designs

Selina Volsa, David Lewetz, Vinka Mlakic, Chiara Bertagnoli, Samantha Hochstöger, Martina Rechl, Hannah Sertic, Bernad Batinic, and Stefan Stieger

# Overview of the Open-Source Android Application

In the main article we described the most important configuration functions our open-source Android application provides to make the MetaMotionR wearables usable for ESM studies. In this section of the supplement, we additionally want to give a more detailed overview of the workflow and capabilities of the application.

## Connection Interface

The application’s main screen (shown in Figure S1 on the left) allows a user to connect to a nearby wearable via Bluetooth. If this option is selected, the next screen will show a list of available wearable devices, identified by their MAC address (shown in Figure S1 on the right). The application also shows the signal strength and has the option to automatically connect to a device if this signal strength is above a certain threshold. This allows to physically position the device one intends to configure next to the smartphone to establish a connection.

Figure S1.

Main Screen (Left) and Device Selection Screen (Right) of Configuration Application


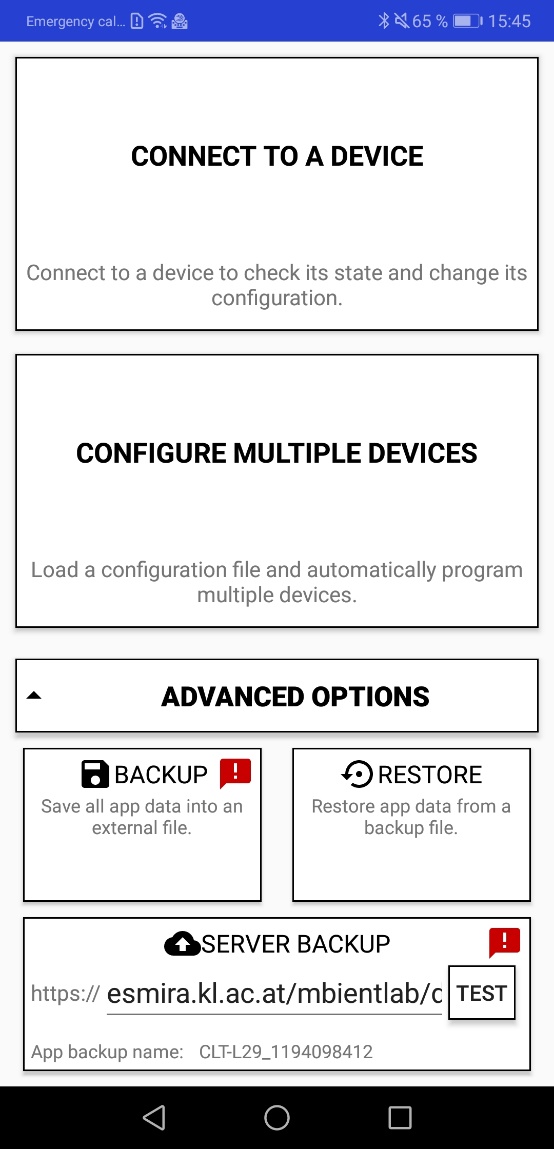

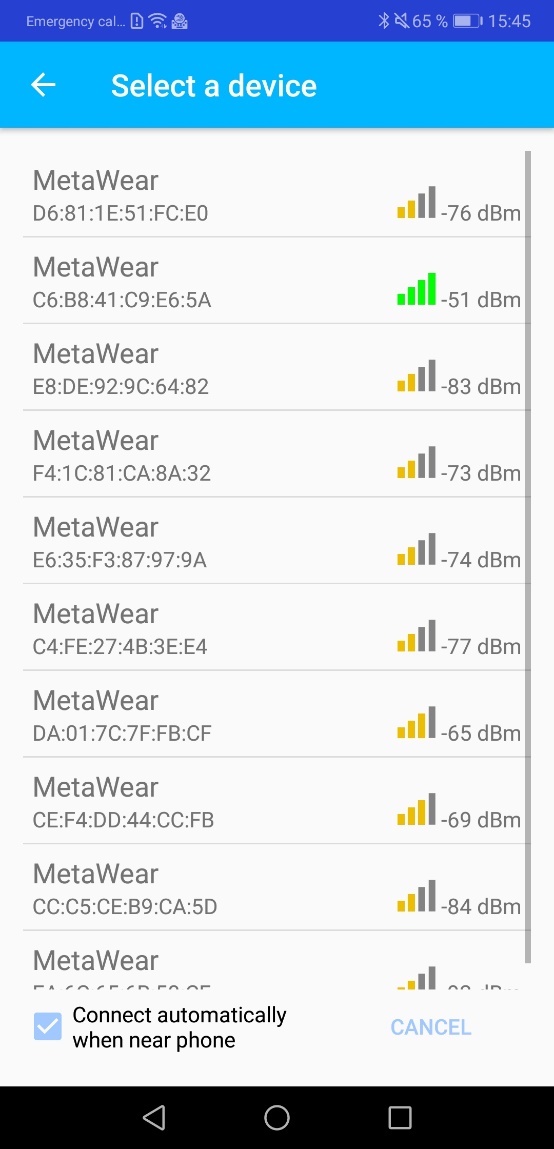


Beyond establishing a connection to a single device, the application also offers the possibility to send a saved configuration to multiple devices simultaneously. This configuration must first be created on a single device and then saved. This process will be explained in the section concerning the administration of individual devices below.

An important aspect concerning the device configuration is the fact that most data concerning configurations is stored on the smartphone itself. Programmed configurations cannot be read out of a wearable. Furthermore, the wearable only keeps internal timer values relative to the time of programming it. Therefore, a wearable device’s configuration, alongside information such as the time of programming are stored in the smartphone application’s data. This means that corruption or loss of the smartphone could potentially render data from programmed wearables useless, as their log times cannot reliably be associated with real times anymore. To remedy this, the smartphone application allows the creation of backups. A backup can later be used to restore a smartphone application to the stored state, recovering the configuration parameters of all saved devices. This also allows to use different smartphones for programming a wearable and downloading data from it, as long as the application state was transferred between the two smartphones. In order to make the backup process more reliable, the application also offers the option to directly send the application state backup to a server (note that this requires administration rights on that server). Although this sounds impractical, it is also an important data safety option. Only the study investigator who is in possession of the smartphone used to program the wearable or is in possession of the configuration file can readout the data from the wearable reliably.

## Administering a Device

When a connection is made with a device, the application will first check whether the device has previously been programmed by another smartphone. If so, it will display a warning, and give the option of clearing all data from the wearable, as reconfiguring would make existing data invalid. Furthermore, the application will detect the firmware version of the device and display a warning if it is outdated (see Figure S2).

Figure S2.

Warning Screens Displayed Upon Connection in the Configuration Application (if Applicable): Warning Regarding Configuration by Other Smartphones (Left) and Warning Regarding Firmware Version (Right)


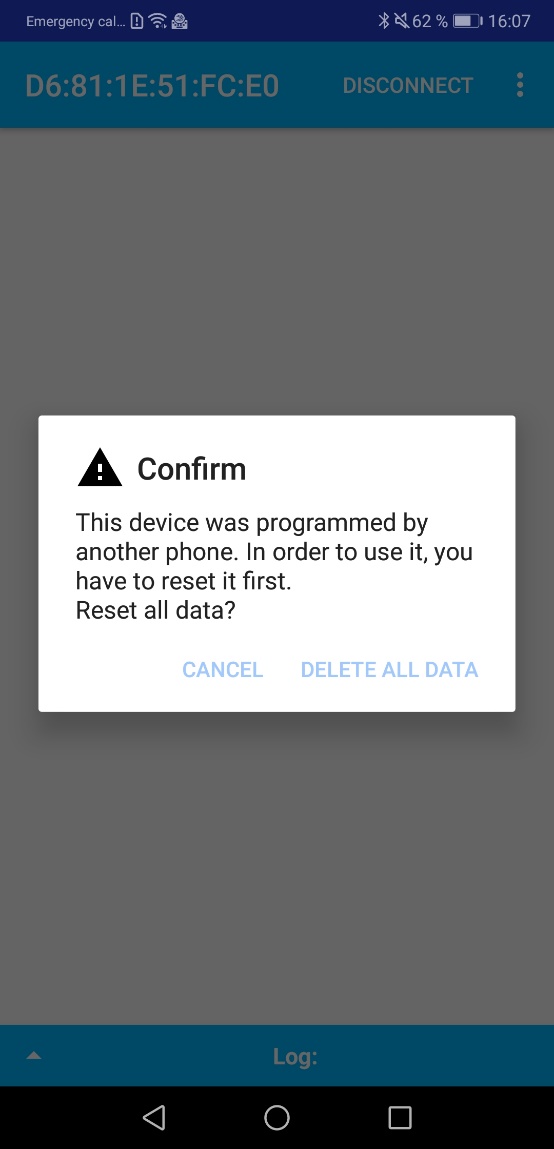

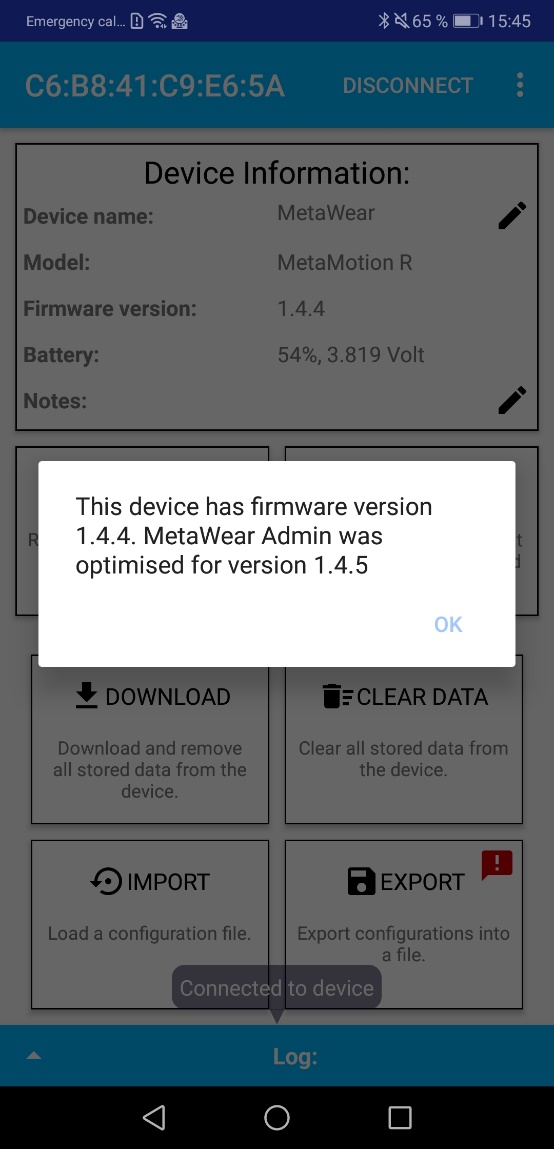


The device overview screen (shown in Figure S3, on the left) displays some information about the device (i.e., device name and model, the firmware version, the current battery percentage, and optional notes) as well as options to interact with the device. The “new” button will reset the device and enter the configuration process, explained in detail below. The “view” button shows the configuration state the application currently associates with that wearable (i.e., the information is taken form the information stored on the smartphone, not read out from the wearable). The “download” button starts the download process. This will open a popup (as shown in Figure S3, on the right) that shows the available types of data. If the device was configured to log both, the press count as well as the acceleration, this pop-up will offer the option to create an additional file combining these two data sources for easier use in statistical software packages. Once the user proceeds from this pop-up, the smartphone will download all data from the wearable and store the files within the smartphone’s downloads directory. Note that this process also deletes all stored data on the wearable, i.e., it is not repeatable with the same data. The device overview screen also allows to directly delete all data on the device via the “clear data” button. The “import” and “export” buttons allow to store and load device configurations to and from configuration files on the smartphone. This makes it also possible to share configurations between smartphones or to reuse a wearable’s configuration for other wearables. Lastly, a log screen (not shown in Figure S3) accessible at the bottom of the screen shows details of all interactions between the application and the device.

Figure S3.

Device Overview Screen (Left) and Download Pop-Up (Right)


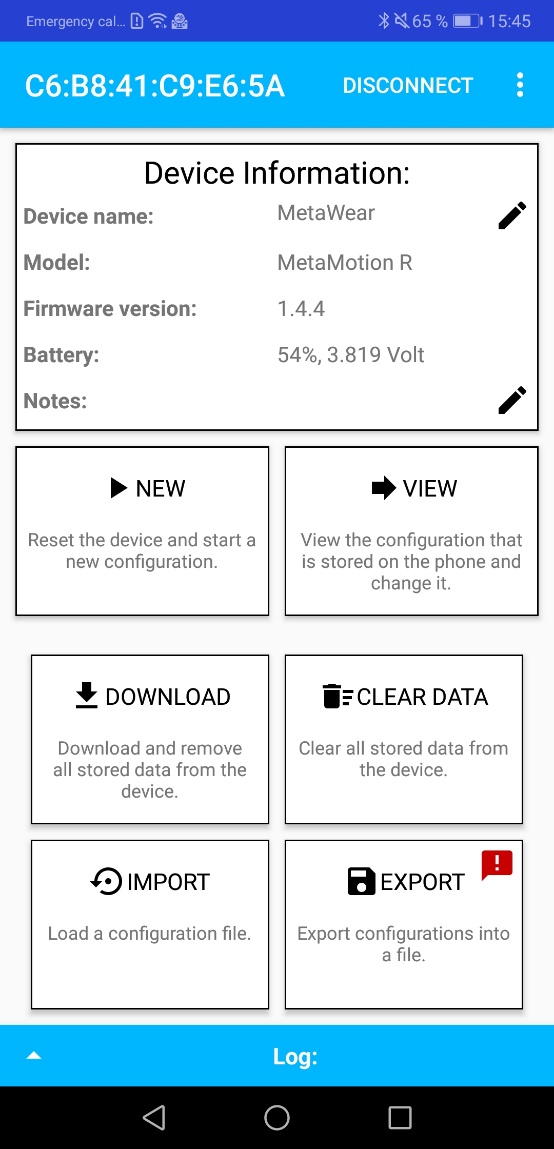

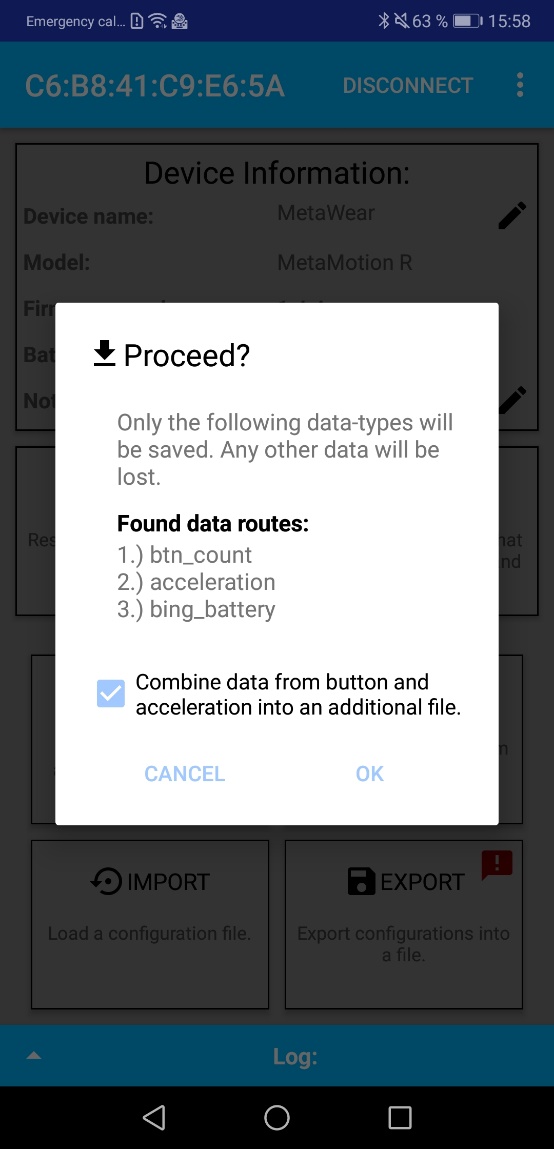


### Device Configuration

The device configuration interface consists of three separate tabs: Bings, button, and battery (see Figure S4). The bings tab allows the creation of timed bings that may trigger certain device behaviors and log entries. Figure S4 shows the bing configuration tab, both in its initial state without any bings, as well as with multiple configured bings. Once populated with bings, the application shows their configuration with several icons. One important aspect of the software is that both reminders (i.e., reoccurring bings, if there was no reaction to a bing) and pseudo-random timers are possible but allow only a single configuration each. For example, if a reminder is set to repeat up to three times every 15 minutes, then this reminder may be attached to any other bing, but another reminder with different parameters is not possible. As soon as a reminder or a random timer are configured, the application shows this configuration within the bings tab.

Figure S4.

Bing Configuration Tab, Both Empty (Left) and With Multiple Bings Configured (Right)


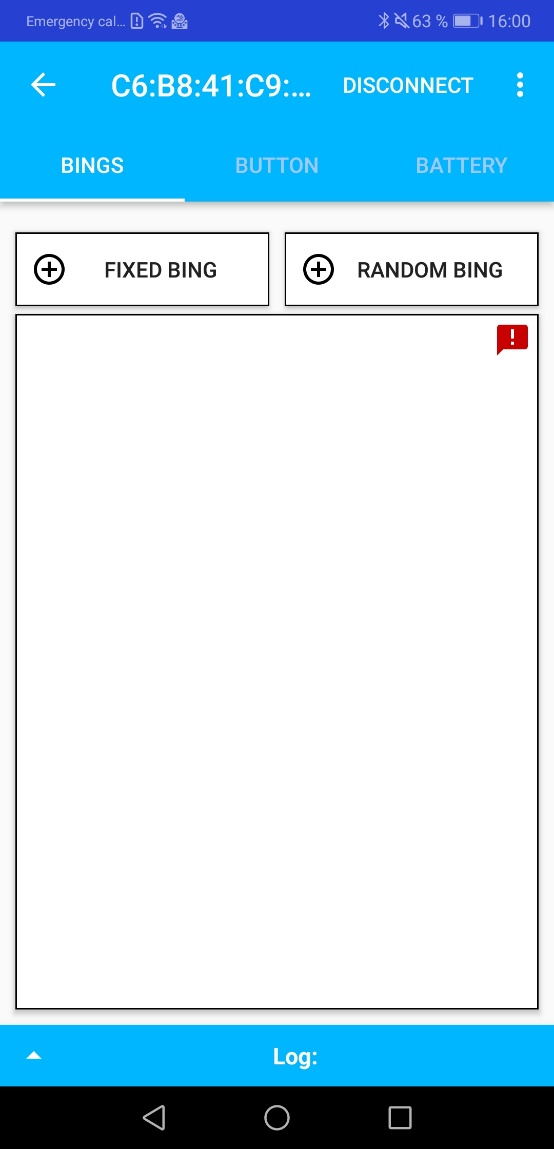

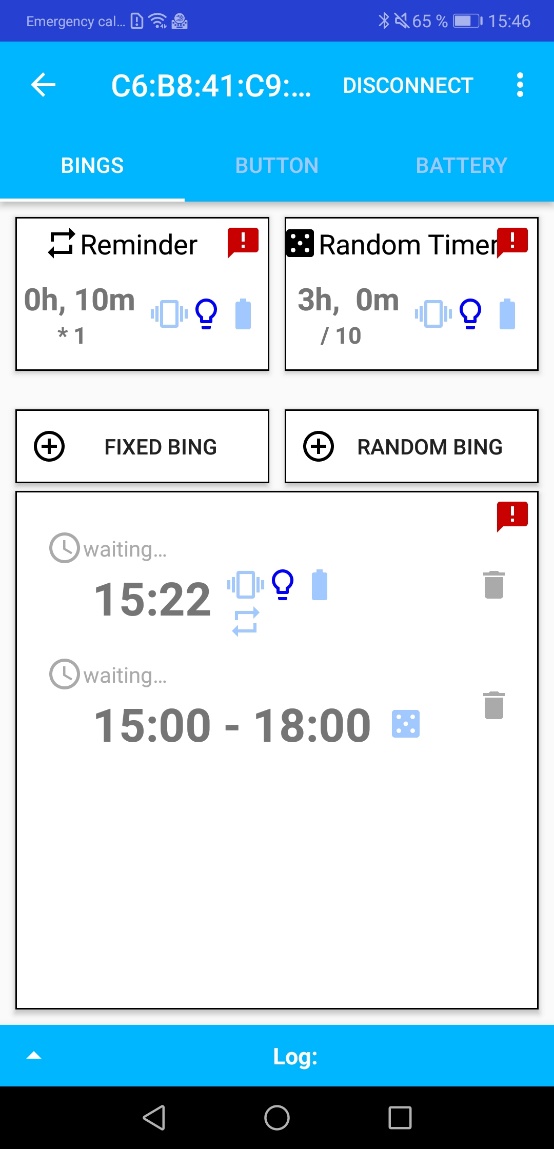


To add a fixed bing, the corresponding button can be pressed to open a configuration pop-up (see Figure S5). This pop-up allows to set the time the bing should trigger, as well as options for that trigger. The bing may flash the LED in a certain color (red, green, or blue), and may activate the vibration motor for a configurable duration at a configurable intensity. Furthermore, the time-stamp and battery state may be logged, to create a record of when the bing happened. On the next page within the pop-up, a reminder can be enabled. If the reminder is not yet configured, this page also allows to configure the behavior of the reminder, i.e., how long the repeat intervals should be, and up to how often the reminder should be repeated. Furthermore, the reminder has the same feedback and logging options as the main bing (i.e., LED, vibration, and battery logging). Once in use, the reminder will trigger at the set interval up to the configured number of repeats, unless the wearable receives user input, which will cancel further reminders for that bing. Once the bing and the optional reminder are fully configured, the “save” button sends that configuration to the wearable instantly.

Figure S5.

Fixed Bing Configuration Pop-Up


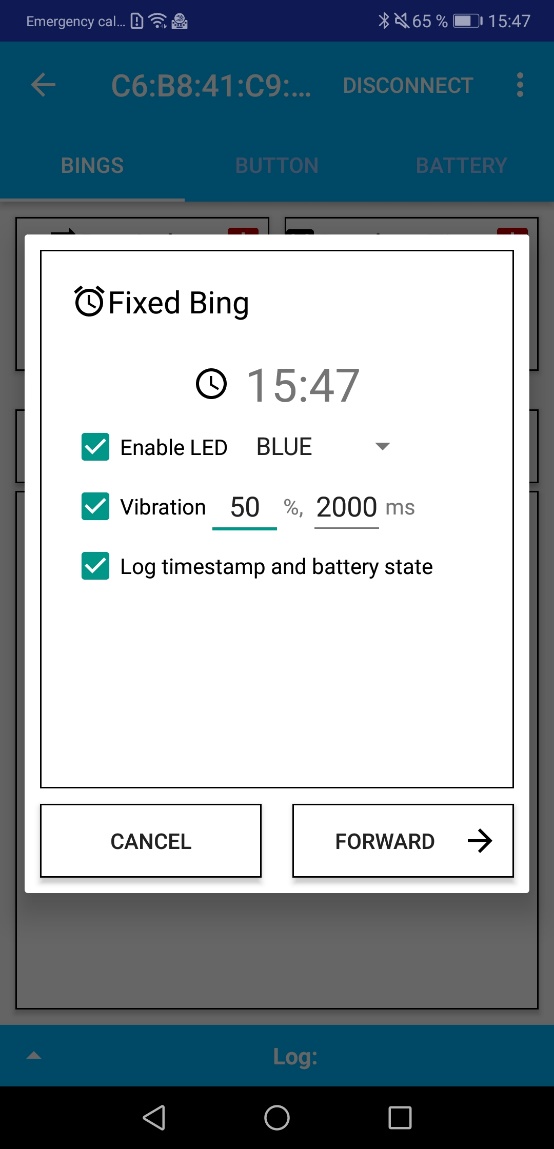

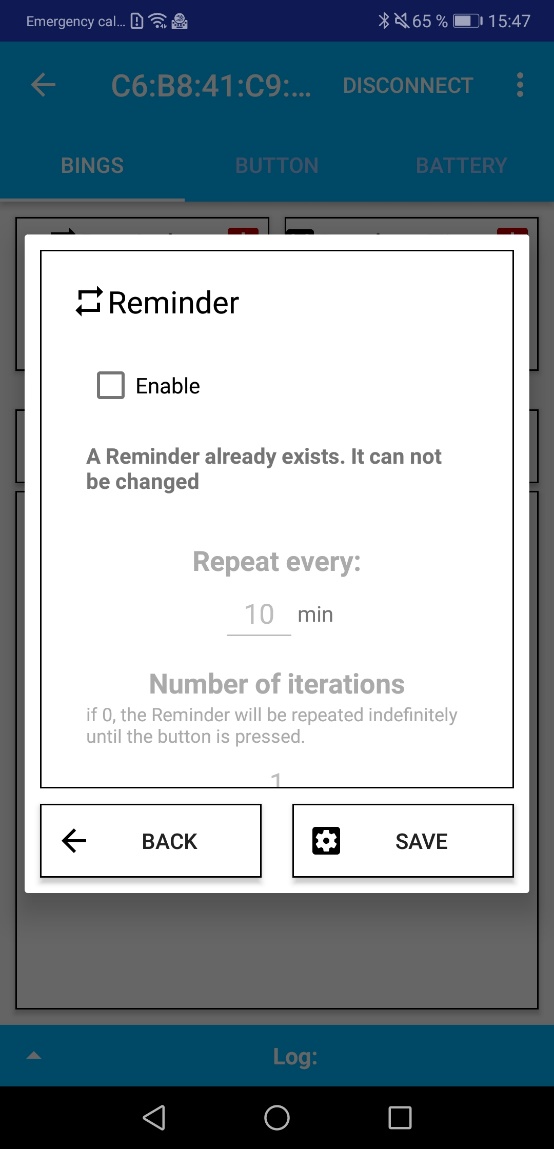


To add a randomized bing, the corresponding button will open a pop-up similar to that for the configuration of fixed bings (see Figure S6). However, beyond the same options for feedback and logging (i.e., activation of LED and vibration motor, logging of battery state), this screen allows to set up the time window of the random timer, if it has not been set up yet. It also allows to set a fixed time, similar to the fixed bing, which will act as the start of the time window in which the bing may trigger. To configure the time window, the user can set the length of the time window in minutes, as well as the number of possible trigger time points this interval should be divided in. For example, if the randomized bing is set to start at 9 a.m., with a duration of 60 minutes and 12 possible time points, then the bing can trigger in five-minute intervals between 9 a.m. and 10 a.m. A second page on this pop-up allows to set up the reminder. This page is identical to the one mentioned above for the fixed bing.

Figure S6.

Randomized Bing Configuration Pop-Up


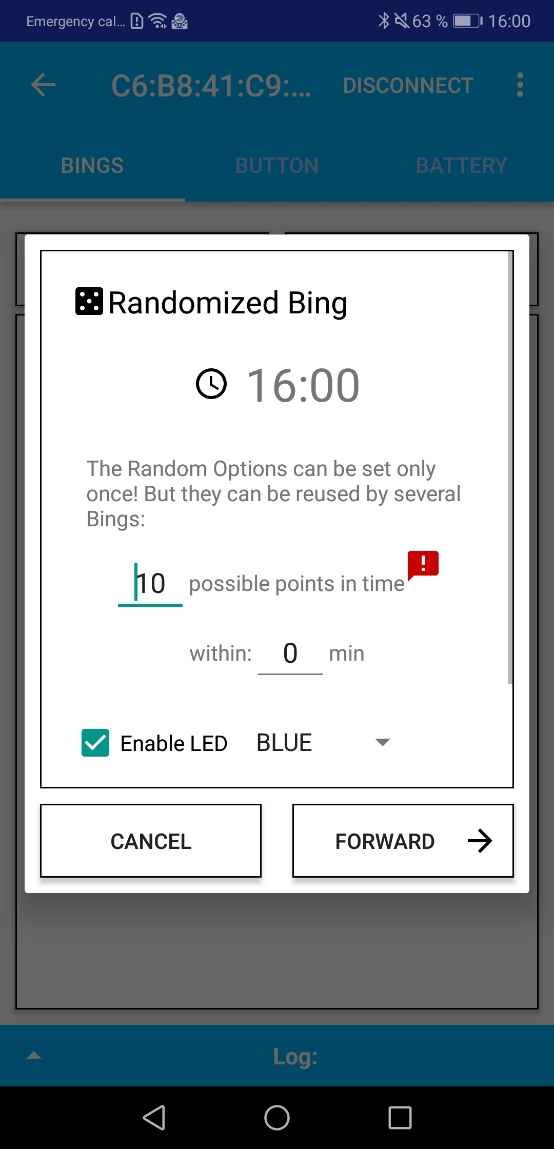

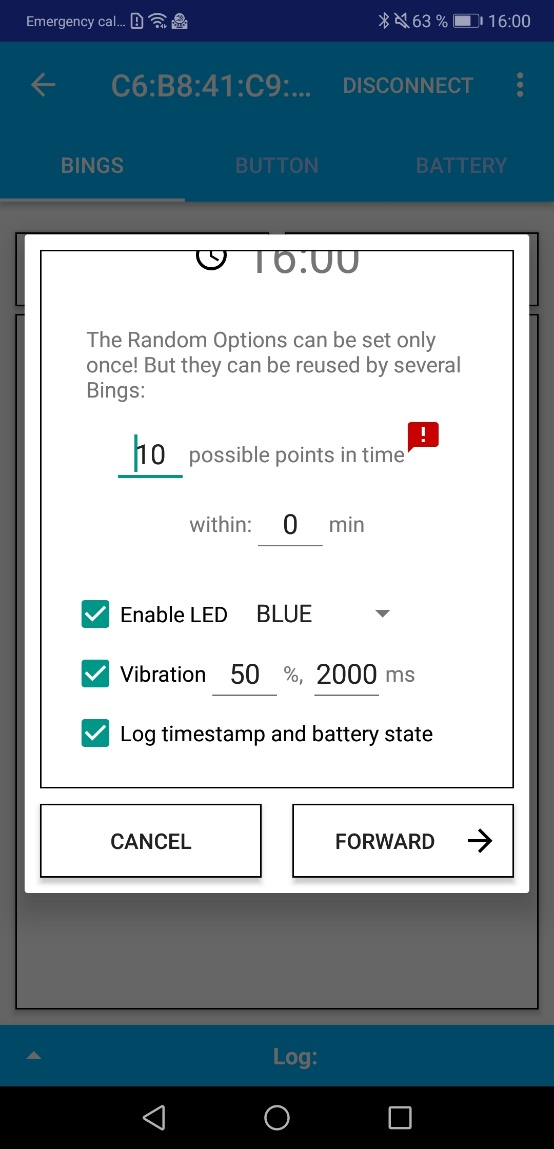


The button configuration tab shows the current configuration of the wearable’s button. Configuring it opens a configuration pop-up, as with bings. Figure S7 shows the button configuration tab, as well as the first screen of the configuration popup. At the bottom of the pop-up the user has the option of setting up the button configuration to be restored on a device reboot. In case the device loses power or is otherwise rebooted, it can restore the button to the set configuration. However, note that in such an event the internal timer will reset, and bing configurations are lost, and only the button behavior is restored. In the logging screen of the configuration pop-up the user can set what should be logged. The screen allows to choose whether or not to log data from the acceleration sensor, as well as one of three button behaviors. The default behavior (i.e., none of the two options selected) simply logs the time of button presses. The other two options are to either log the duration of button presses or the number of consecutive button presses in a three second time window. Depending on what option is chosen, the button press feedback has different options. The default button logging behavior allows to set the behavior of the vibration motor (as before, duration and intensity), as well as an option to blink the LED in one of three colors (red, green, or blue). The press-duration logging behavior has the same options but expands these by the option to activate the LED for the duration of the button press, instead of a single blink (see Figure S7). The option to count the number of button presses has the most extensive configuration options due to its specific behavior. When pressed, the device will start a three-second timer. If the button is pressed again before the end of this timer, the button count is incremented, and the timer reset. If instead the timer runs out the measurement is complete. Therefore, this behavior has two feedback configurations, as seen in Figure S8, on the left. One configuration is for the button presses themselves, the other for the feedback on the three-second timer timing out. The button press feedback can include vibration (with configurable duration and intensity), and either let the LED light up in a specific color or let it cycle through the available colors in a set pattern. This last option can help to further separate the feedback from different consecutive button presses. The timeout feedback can consist of an activation of the vibration motor (with configurable duration and intensity) as well as an activation of the LED in a specific color. Lastly, for all three button logging options the feedback can optionally be replaced in the case the battery runs low (shown in Figure S9, on the right). This changed feedback can consist of vibration and an LED activation in a specific color. The threshold percentage under which this behavior change should occur, can also be configured. This can alert users of the wearable that the device needs to be recharged.

Figure S7.

Button Configuration Tab With Icons Indicating Current Configuration (Left) and First Screen of Button Configuration Pop-Up (Right)


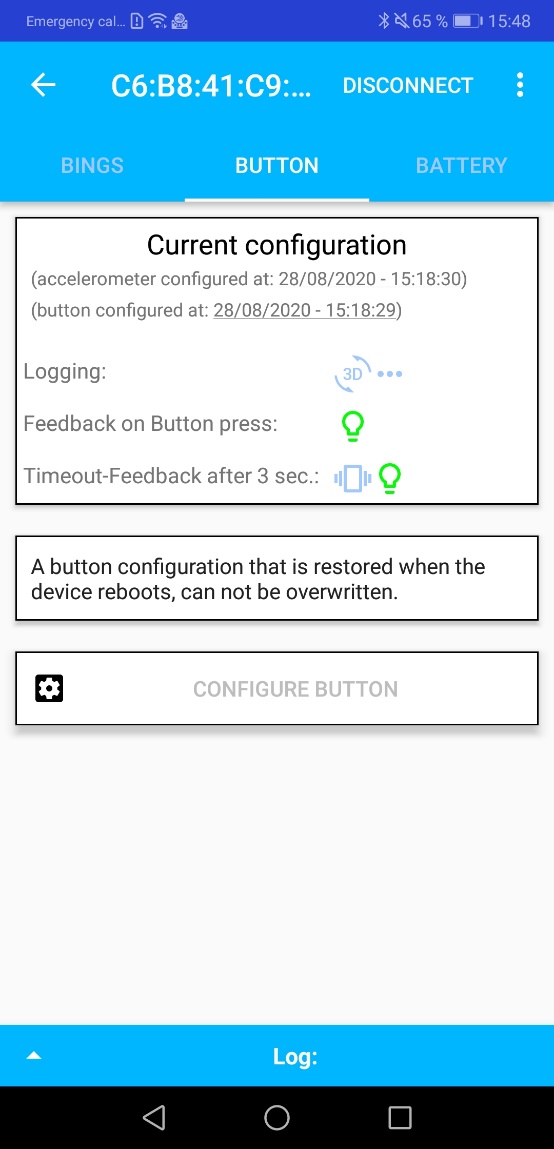

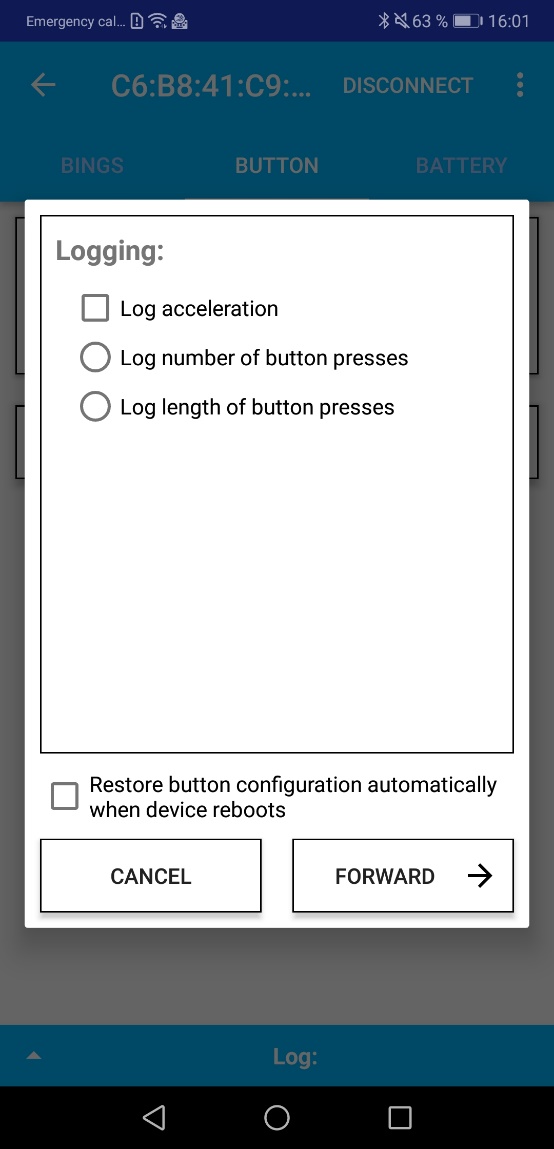


Figure S8.

Button Press Feedback Configuration Options for Default Logging Behavior (Left) and Duration Logging Behavior (Right)


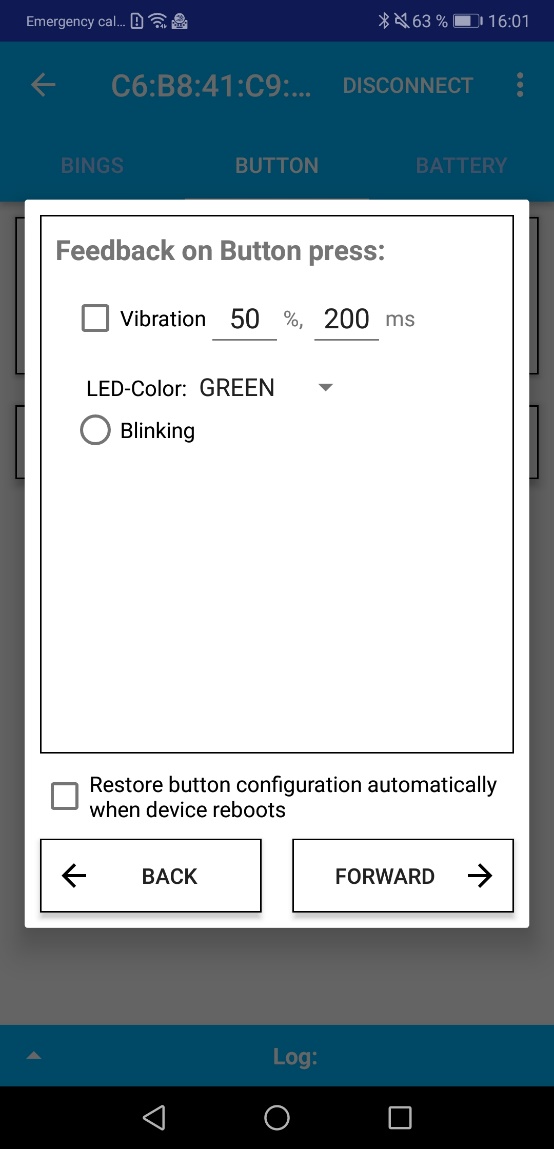

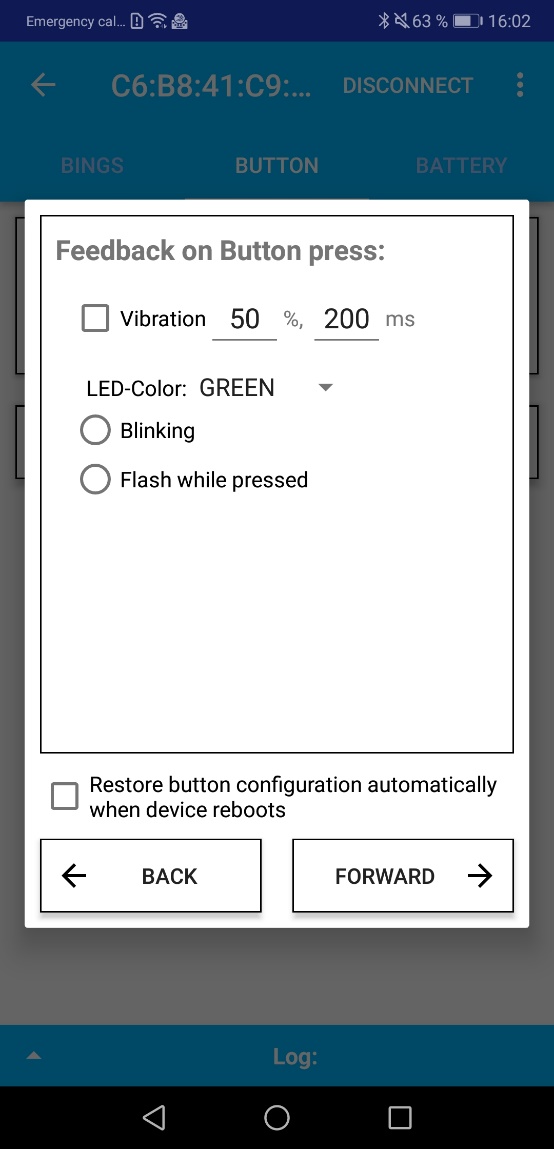


Figure S9.

Button Press Feedback Configuration Options for Logging of Number of Button Presses (Left) and for Low Battery (Right)


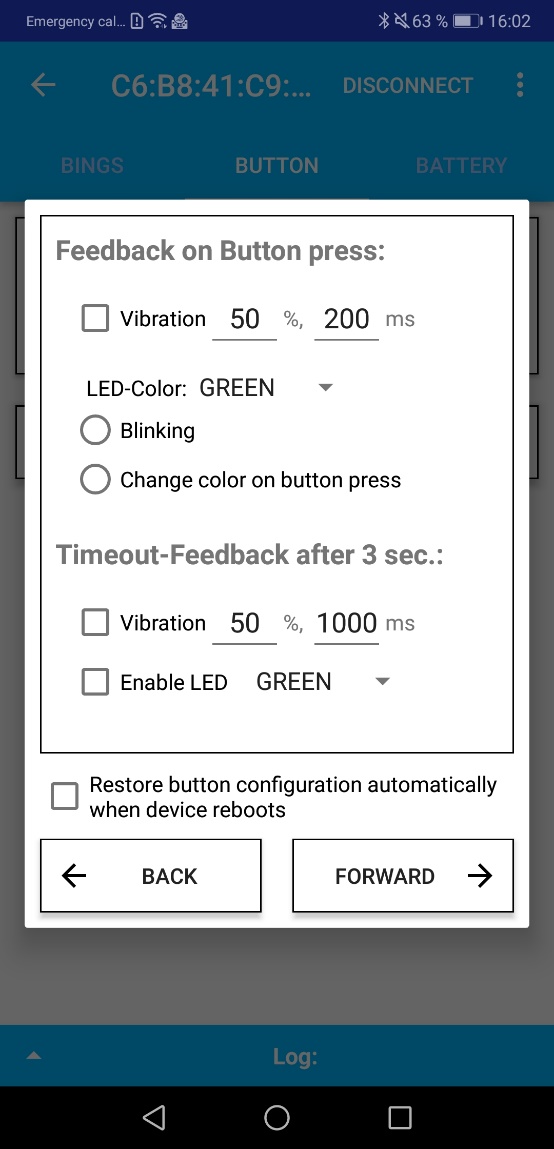

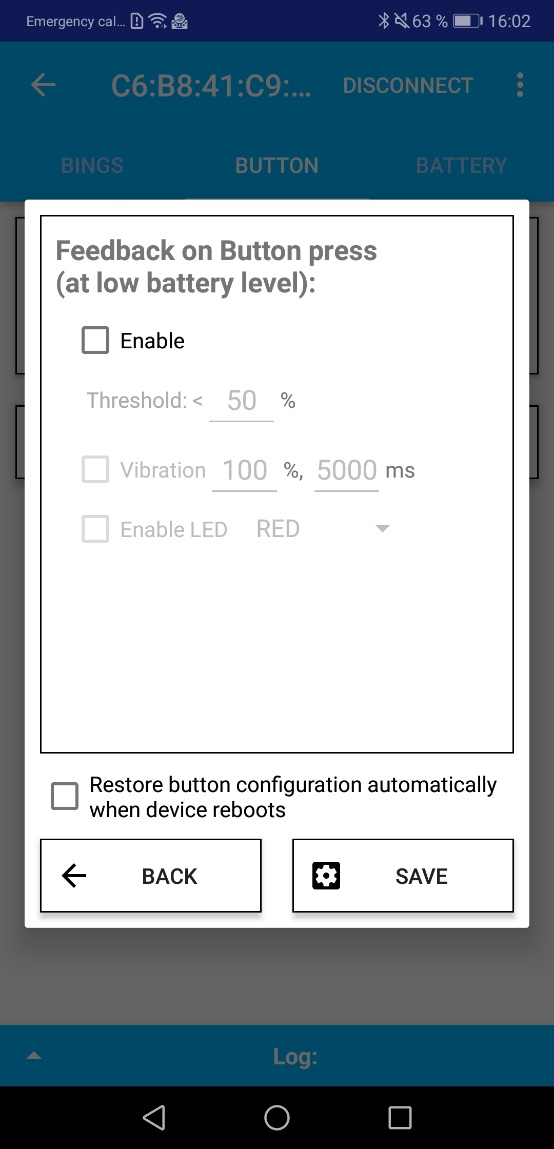


The last configuration tab is the battery configuration tab. It shows options to configure two types of battery-related feedback (see Figure S10). The reboot feedback occurs when the device reboots. It can consist of an LED activation in a configurable color (red, green, or blue) as well as an activation of the vibration motor (with configurable duration and intensity). It may also repeat at configurable intervals. As the device loses all timing related configuration (i.e., timers are lost and the internal clock used for log time stamps is reset), this can serve as feedback for study participants that the device is not working properly anymore, and that they may need to interrupt the study protocol. The other type of feedback is feedback for charging. This allows LED feedback as well as vibration feedback when the device has started charging.

Figure S10.

Battery Configuration Tab


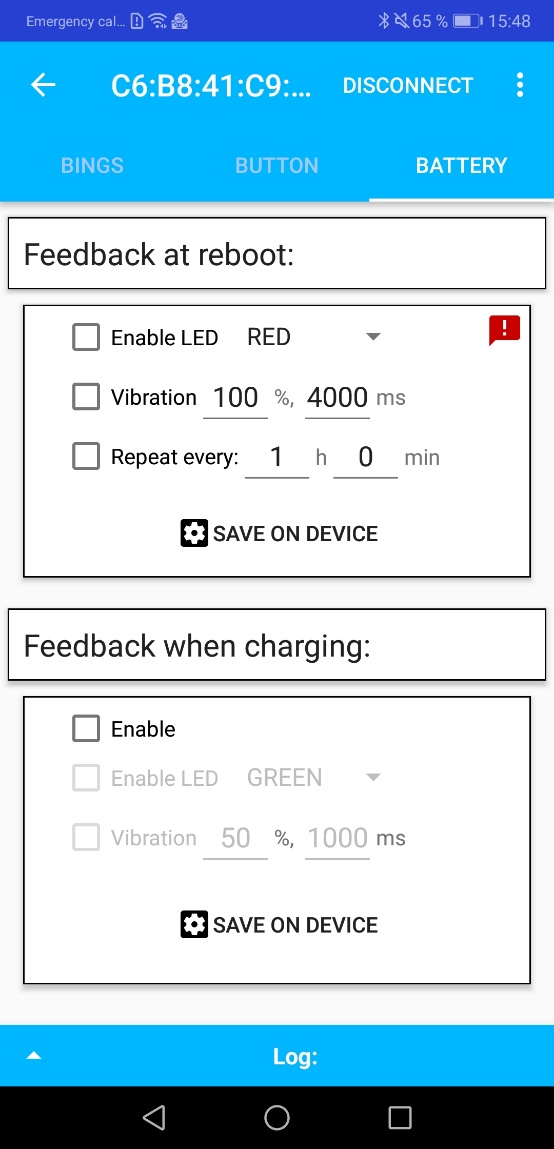


# Third Battery Test

We performed a third battery test, akin to Pilot Studies 1 and 2. The aim of this repeated test was to compare the wearables’ battery life across different design complexities. The original two tests only used two bings per day, each with vibration and light. However, in a design with more signals these bings would be triggered more often and could therefore consume more power, more quickly draining the battery. We therefore repeated the test with nine more wearables. These devices (including their batteries) were roughly four years old at the time of the test.

The wearables were divided into three groups, each containing three devices. The first group was set up with two bings per day, similar to the prior two tests. The second group was set up with five bings per day (the maximum number possible due to memory constraints), and five reminders. This meant that both the vibration motor as well as the LED should be activated 30 times each day. The third group was set up the same way but would receive button presses whenever possible (thereby interrupting the reminders and resulting in a minimum of five bings per day. The buttons of the third group were pressed up to four days per week.

Figure S11 shows the battery life for all devices. Device 1, in the first group, did show an unusually low runtime of just under ten days. However, while groups two and three did show shorter runtimes than the remaining two devices from group one, they still consistently managed to stay active for over 20 days. There seem to be no obvious differences between the second and third group.

Figure S11.

Battery Runtime of Third Battery Test.


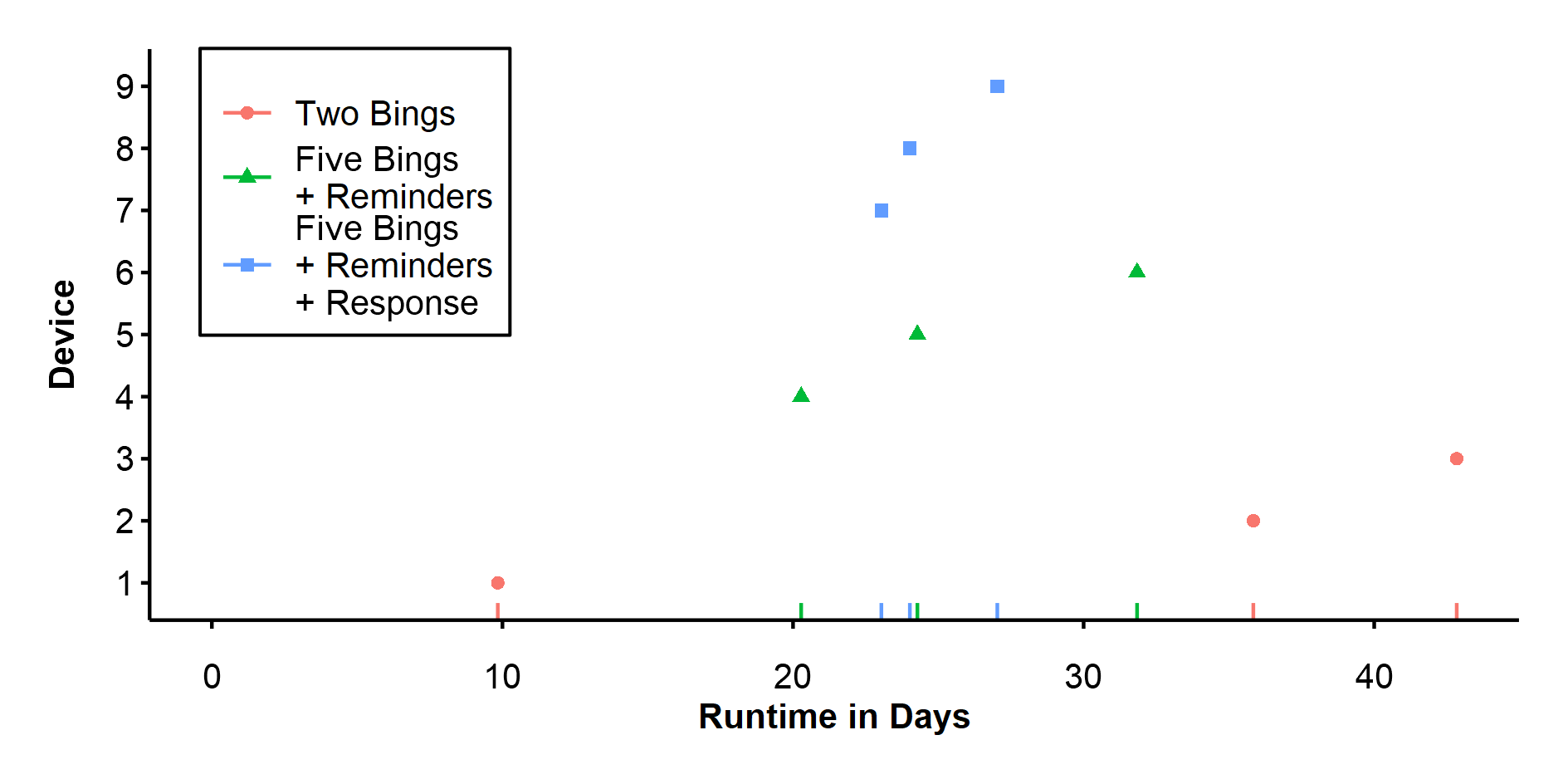


# Angle Measurements from Pilot Study 6 Separated by Gender

Table S1.

Mean Angles for Each Target Angle From Pilot Study 6, Separated by Gender

| Target Angle | Overall | Male | Female |
| --- | --- | --- | --- |
| 0° | -0.25 (8.91) | 0.04 (5.74) | -0.33 (9.22) |
| 45° | 40.89 (11.66) | 44.25 (8.72) | 40.54 (11.95) |
| 90° | 77.60 (8.59) | 81.04 (6.17) | 77.14 (8.72) |

*Note.* Standard deviations in parentheses.

# Model Specification of Multilevel Analysis in Pilot Study 6

The following model was used in the multilevel analysis in Pilot Study 6, with observations (Level 1) nested in :

$$ScalesDifference = PAS - VAS$$

Level 1: $\mathrm{ScalesDifferenc}e_{ti}=\pi_{0i}+\pi_{1i}VAS+e_{ti}$

Level 2: $\pi_{0i}=\beta_{00}+r_{0i}$

Level 2: $\pi_{1i}=\beta10+r_{1i}$

The model was estimated as a linear model using restricted maximum likelihood.

Original Definition of Belly Laughs and Fits of Laughter

The German definition of a belly laugh presented to participants was: “Mit Bauchlachen ist ein echtes, lautes, Lachen gemeint, das von Herzen kommt. Es bringt den ganzen Körper in Bewegung, besonders Bauch und Brust. Es ist gekennzeichnet durch rhythmische Bewegungen des Zwerchfells und wird meistens automatisch ausgelöst.“

The German definition of a fit of laughter presented to participants was: “Unter einem Lachanfall versteht man heftiges Lachen über eine längere Zeit hinweg, begleitet von Tränen und dem Gefühl, nicht aufhören zu können.“

# Preregistered Analysis for Hypothesis 1 From Main Study

Originally, the Main Study’s preregistration (found at <https://osf.io/yjgfu>) suggested that Hypothesis 1 would be tested using a *t*-test. We deviated from this analysis plan as we later realized that a *t*-Test would not be appropriate for nested count data. We therefore opted to use generalized linear mixed models with a negative-binomial distribution. This also allowed us to include several other motivational covariates. However, we wanted to ensure that this deviation would not alter our main finding. Therefore, we chose to also test Hypothesis 1 using both a *U*-test as well as a generalized linear mixed model using only the device as a predictor.

## Using a *U*-Test

To achieve independent data, we first aggregated the daily event counts to the subject level using means (i.e., every data point would represent the average daily laughter frequency of a participant). However, due to the use of count data the resulting distributions exhibit substantial skew, necessitating the use of a non-parametric test. Therefore, we used a Mann-Whitney *U*-test to assess Hypothesis 1 here. This *U*-test indicated the difference between the wearable group (*Md* = 2.96, *M* = 4.88, *SD* = 5.13) and the smartphone group (*Md* = 1.08, *M* = 1.53, *SD* = 1.42) to be statistically significant (*U* = 864, *p* < .001).

## Using a Generalized Linear Mixed Model With a Single Predictor

As we included variables in the model that originally were not in the preregistration, we also checked whether the result persisted when including only the group variable (i.e., the used device) in the model. This would result in the following model specification:

Level 1: $\text{log}\left( EventCount_{ti} \right)=\pi_{0i}+e_{ti}$

Level 2: $\pi_{0i}=\beta_{00}+\beta_{01}Wearable+r_{0i}$

Table S1 shows the result of this model. In this model, the device variable also shows a significant difference. Converted to a factor, the coefficient *B* = 1.216 corresponds to a 3.37 times higher count in the wearable group. This coefficient and factor are similar to what was found in the Main Study.

Table S2.

Results of Generalized Linear Mixed Model Using Only Group Predictor

|  | Fixed Effects | | | | | | Random Effects | |
| --- | --- | --- | --- | --- | --- | --- | --- | --- |
|  | Coeff. | *B* | CI | *SE* | *z* | *p* | Coeff. | *SD* |
| Intercept | β_00_ | 0.059 |  | 0.112 | 0.526 | .599 | r_0i_ | 0.897 |
| Wearable | β_01_ | 1.216 |  | 0.166 | 7.319 | < .001 |  |  |
| *N_par_* = 134, *N_obs_* = 3,054, ICC = .58, *φ* = 0.25 | | | | | | | | |

*Note.* Reference category for wearable was smartphone, ICC = Intra-Class Correlation of the nullmodel.

## Conclusion

Both approaches show significant differences between the two groups, supporting the robustness of the findings in the Main Study. This lends confidence that the deviations from the preregistration do not impact these findings.

# Analysis of End-of-Day Data From Main Study

As part of the Main Study’s protocol, participants filled out a daily end-of-day questionnaire. This questionnaire allowed participants to account for laughter events they believe they had forgotten throughout the day. Our initial idea was to use this data to supplement the event count data. Therefore, we first analyzed the two separately to ensure compatibility of two data sources. This analysis used the same model specification as shown in the Main Study.

Table S2 shows the results of this analysis. Noteworthy here is the overall lack of significant predictors, save for the daily compliance. However, we expect the factual average laughter frequency to be constant across groups and throughout the course of the study. Therefore, we would expect to see similar results here as can be seen in the results of the Main Study concerning the count data. If participants in the smartphone group log less of the laughter events they experience, we would expect them to compensate in the end-of-day questionnaire and enter a higher count of “forgotten laughter events”. Nonetheless, the wearable predictor in this analysis has no significant influence, meaning that the number of forgotten events entered in the end-of-day questionnaire did not differ between the two groups. Furthermore, in the count data both groups showed a decline in their daily count throughout the study duration. This most likely indicates a decline in overall motivation. We would expect this decline in motivation to also show here. This effect could express itself in both directions. Participants could either compensate for their decline in logs by entering bigger numbers of forgotten laughs in the end-of-day questionnaire over the course of the study. On the other hand, they could also be less motivated to keep track of the events they did not enter right away, and thus enter smaller numbers towards the end of the study (because the count they keep is lower) or always enter similar numbers (because they are estimating rather than keeping count). However, the day variable in this analysis shows no significant influence on the entered number.

Table S3.

Results of Generalized Linear Mixed Effects Model for Non-Entered Laughter Estimates

|  | Fixed Effects | | | | | | Random Effects | | |
| --- | --- | --- | --- | --- | --- | --- | --- | --- | --- |
|  | Coeff. | *B* | *CI* | *SE* | *z* | *P* | Coeff. | *SD* | |
| Intercept | $\beta_{00}$ | 0.618 | 0.045; 1.191 | 0.292 | 2.115 | .034 | $r_{0i}$ | 1.143 | |
| Within-person |  |  |  |  |  |  |  |  | |
| Day | $\beta_{10}$ | 0.003 | -0.011; 0.016 | 0.007 | 0.393 | .694 | $r_{1i}$ | 0.042 | |
| Compliance Day | $\beta_{20}$ | -0.248 | -0.456; -0.040 | 0.106 | -2.339 | .019 | $r_{2i}$ | 0.437 | |
| Between-person |  |  |  |  |  |  |  |  | |
| Compliance General | $\beta_{01}$ | -0.123 | -1.161; 0.914 | 0.530 | -0.233 | .816 |  |  | |
| Missed Days | $\beta_{02}$ | -0.010 | -0.064; 0.044 | 0.028 | -0.352 | .725 |  |  | |
| Wearable | $\beta_{03}$ | -0.004 | -0.417; 0.409 | 0.211 | -0.018 | .986 |  |  | |
| Wearable * Day | $\beta_{04}$ | <0.001 | -0.020; 0.020 | 0.010 | 0.016 | .987 |  |  | |
| *N_par_*= 134, *N_obs_*= 3,054, ICC = .53, *φ* = 0.94 | | | | | | | | |  |

*Note.* Reference category for wearable was smartphone, ICC = Intra-Class Correlation of the nullmodel.

Overall, these results suggest that participants might have estimated a number rather than tracked the instances of events they could remember but had not entered right away. Due to missing the influences one would expect, we believe this estimation is likely to be biased (e.g., memory bias). Furthermore, as there effectively are no group differences, adding these data to the count data would be similar to adding a constant factor. For these reasons we decided to not use these data for the analysis in the Main Study.

# Model Diagnostics of Generalized Linear Mixed Model From Main Study

We performed model diagnostics for the model presented in the Main Study. We used the *DHARMa* package in *R* (Hartig & Lohse, 2022). Figure S11 shows the diagnostic outputs. The Kolmogorov-Smirnov test did reach significance, which is, however, not surprising considering the size of the dataset (3,054 data points). Neither visual inspection of the plots nor other tests show problems with the model.

Figure S12.

Diagnostic Output of DHARMa Package for the Model From Main Study


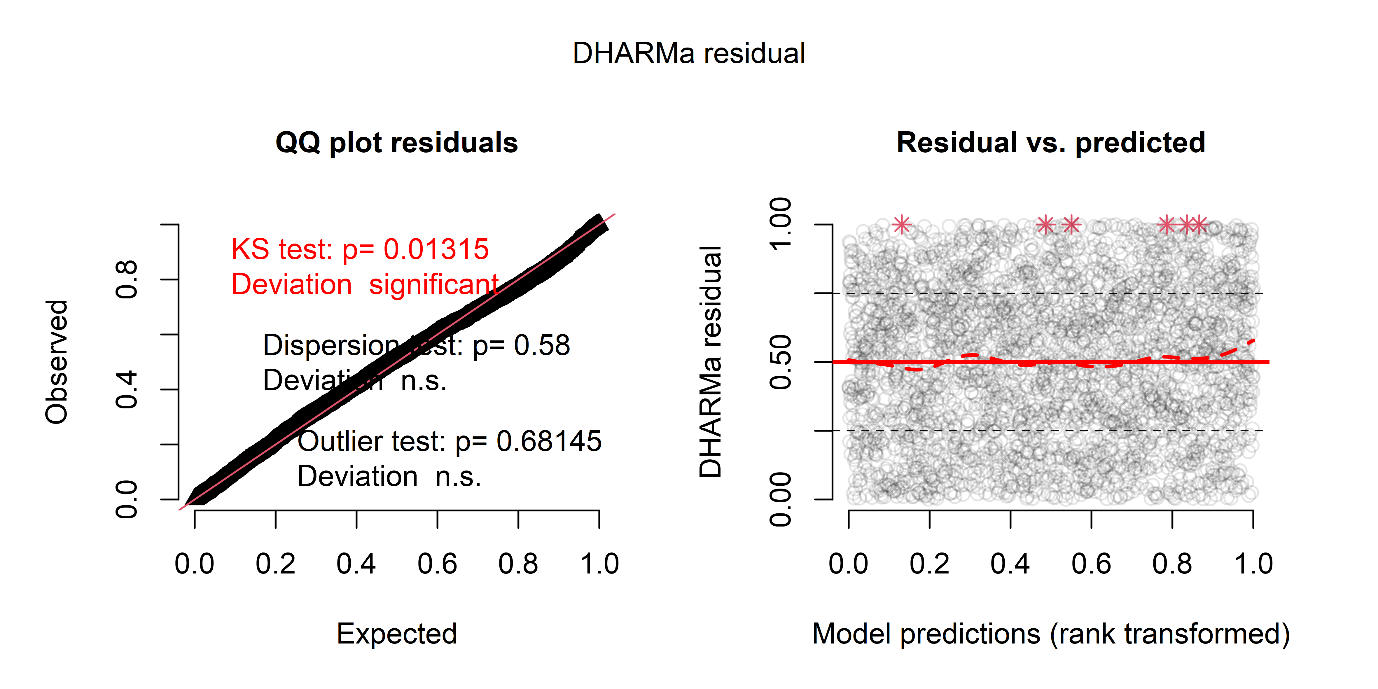


We further compared the density of the data to predicted values as a predictive check (shown in Figure S12). This shows that the masses of the model predictions largely overlap with most of the mass of the data. The model seems to have a slightly higher location, moving from the mode of 0 found in the data to around 1. The model also shows a small peak at around 7 that is not found in the data. However, in the region from 10 to 15, the model underestimates the mass present in the data. Comparing parameters of the data (*M* = 3.28, *SD* = 6.13) with those of the prediction (*M* = 3.15, *SD* = 4.01) shows that the model recovers the location relatively well, but underestimates the dispersion. This overall recovery of the mean is especially important as the mean-subject predictions tend to underestimate the means found in the data (see Figure S12).

Figure S13.

Density Plots of Laughter Event Counts and Subject-Specific Predictions From the Model


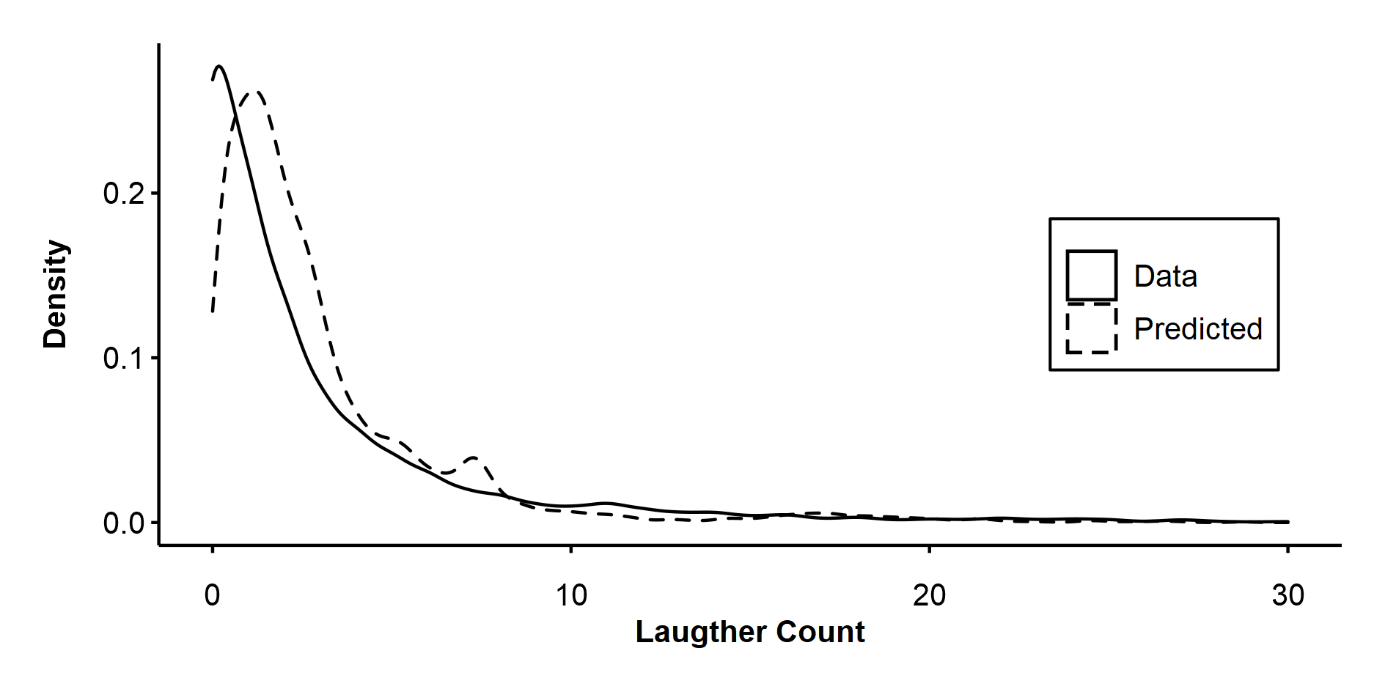

Supplement: Supplementary file 1 — Supplementary file1 (DOCX 2194 KB) [file 13428_2023_2322_MOESM1_ESM.docx]
